# Travel-associated lineages and unique endemic antimicrobial-susceptible lineages of *Neisseria gonorrhoeae* predominate in Western Australia

**DOI:** 10.1099/mgen.0.000969

**Published:** 2023-03-29

**Authors:** Barakat A. Al Suwayyid, Ethan C. Haese, Shakeel Mowlaboccus, Julie C. Pearson, David M. Whiley, Paul K. Armstrong, Carolien M. Giele, Donna B. Mak, Lisa Bastian, Michael J. Wise, Geoffrey W. Coombs, Charlene M. Kahler

**Affiliations:** ^1^​ The Marshall Centre for Infectious Diseases Research and Training, School of Biomedical Sciences, The University of Western Australia, Crawley, Australia; ^2^​ Ministry of Education, Riyadh, Saudi Arabia; ^3^​ Antimicrobial Resistance and Infectious Diseases Research Laboratory, Murdoch University, Murdoch, Australia; ^4^​ Department of Microbiology, PathWest Laboratory Medicine-WA, Fiona Stanley Hospital, Murdoch, Australia; ^5^​ The University of Queensland Centre for Clinical Research (UQ-CCR), Faculty of Medicine, The University of Queensland, Brisbane, Queensland, Australia; ^6^​ Communicable Disease Control Directorate, Department of Health Western Australia, Perth, Australia; ^7^​ School of Medicine, University of Notre Dame Australia, Perth, Australia; ^8^​ School of Physics, Mathematics and Computing, University of Western Australia, Perth, Australia; ^9^​ Telethon Kids Institute, Nedlands, Australia

**Keywords:** gonococcal epidemiology, antimicrobial resistance, MLST, iPLEX, NG-STAR

## Abstract

In Australia, gonococcal isolates are monitored for antimicrobial susceptibilities. In Western Australia (WA), gonorrhoea notification rates increased by 63 % between 2013 and 2016, with the steepest increase occurring between 2015 and 2016, before stabilizing at this higher baseline between 2017 and 2020. This increased prevalence was associated with antimicrobial-susceptible (AMS) lineages. To understand the provenance of these isolates causing gonorrhoea in WA, whether they were introduced or expanded from endogenous lineages, 741 isolates were collected in 2017 and characterized by both iPLEX typing and whole genome sequencing (WGS). Antibiograms and genocoding of the isolates revealed that AMS isolates were most prevalent in the remote regions, while the urban/rural regions were characterized by antimicrobial-resistant (AMR) isolates. iPLEX typing identified 78 iPLEX genotypes (WA-1 to WA-78) of which 20 accounted for over 88 % of isolates. WA-10 was the most frequently identified genotype in the urban/rural regions whilst WA-29 was the most frequently identified genotype in the remote regions. Genotypes WA-38, WA-52 and WA-13 accounted for 81 % (*n*=36/44) of the azithromycin-resistant *N. gonorrhoeae* (AziR) isolates. A representative isolate of each iPLEX genotype and AMR biotype was whole genome sequenced and analysed using MLST, NG-MAST and NG-STAR, and the novel core genome clustering Ng_cgc_400 typing scheme. Five predominant Bayesian population groups (termed BPG-1 to 5) were identified in the study collection. BPG-1 and BPG-2 were associated with AMS isolates from the remote regions. BPG-1 and BPG-2 were shown to be unique to the remote regions based on a minimum spanning tree against 4000 international isolates. AMS isolates in urban/rural regions were dominated by international lineages. AziR and Cef DS (decreased susceptibility to ceftriaxone) was concentrated in three urban/rural genomic groups (BPG-3, 4 and 5). Azithromycin minimum inhibitory concentrations (0.5–16 mg l^−1^) correlated with the accumulation of *mtrR* mutations or/and the fraction of 23S rRNA C2611T mutated copies. The majority of isolates in BPG-3, 4 and 5 could be correlated with known AMR lineages circulating globally and nationally. In conclusion, the surge in AMS isolates in WA in 2017 was due to importation of international AMS lineages into urban/rural regions, whilst the local AMS lineages persisted largely in the remote regions. Bridging between the urban/rural and remote regions was relatively rare, but continued surveillance is required to prevent ingress of AMR strains/lineages into the remote regions of WA.

## Data Summary

All fastq files and assemblies were submitted to the National Center for Biotechnology Information (NCBI). All data can be found under Bioproject PRJNA868503. Strain-specific details can be found in the Methods under data deposition.

**Impact Statement**

The macro-epidemiology of *Neisseria gonorrhoeae* genetic lineages must be monitored to understand the movement of antimicrobial resistance in communities around the world. However, it is now clear that the prevalence of the antimicrobial resistance in any geographical location is specific for the context of the communities in each region. Western Australia is one of the most remote locations globally and in this study we show that there are local endogenous antimicrobial-sensitive genetic lineages which dominate in remote communities, while the urban areas are characterized by international genetic lineages, both antimicrobial-resistant and antimicrobial-sensitive. This work shows the importance of understanding the macro-epidemiology of *N. gonorrhoeae* for implementing specific interventions based on the local community level.

## Introduction

*Neisseria gonorrhoeae* is the causative agent of the sexually transmitted infection gonorrhoea [1]. Lower genital tract infections in men are commonly symptomatic while, among women, they are typically asymptomatic or minimally symptomatic [2].

The worldwide prevalence of gonorrhoea has increased annually with an estimated 86 million new cases per annum [3], the highest median rate of gonorrhoea occurring in men. The highest regional rate, 52.4 per 100 000 population, occurs in the Western Pacific region which includes Australia [4]. Gonococcal infections in Australia are primarily reported in three distinct populations: (a) urban men who have sex with men (MSM), (b) geographically remote Indigenous community heterosexuals [5, 6] and (c) urban heterosexuals [7]. Molecular typing techniques have shown that in Australia and Western Australia (WA) there are resident gonococcal strains circulating in localized areas and, from time to time, new strains are introduced from international and interstate travellers [8, 9].

**Fig. 1.**
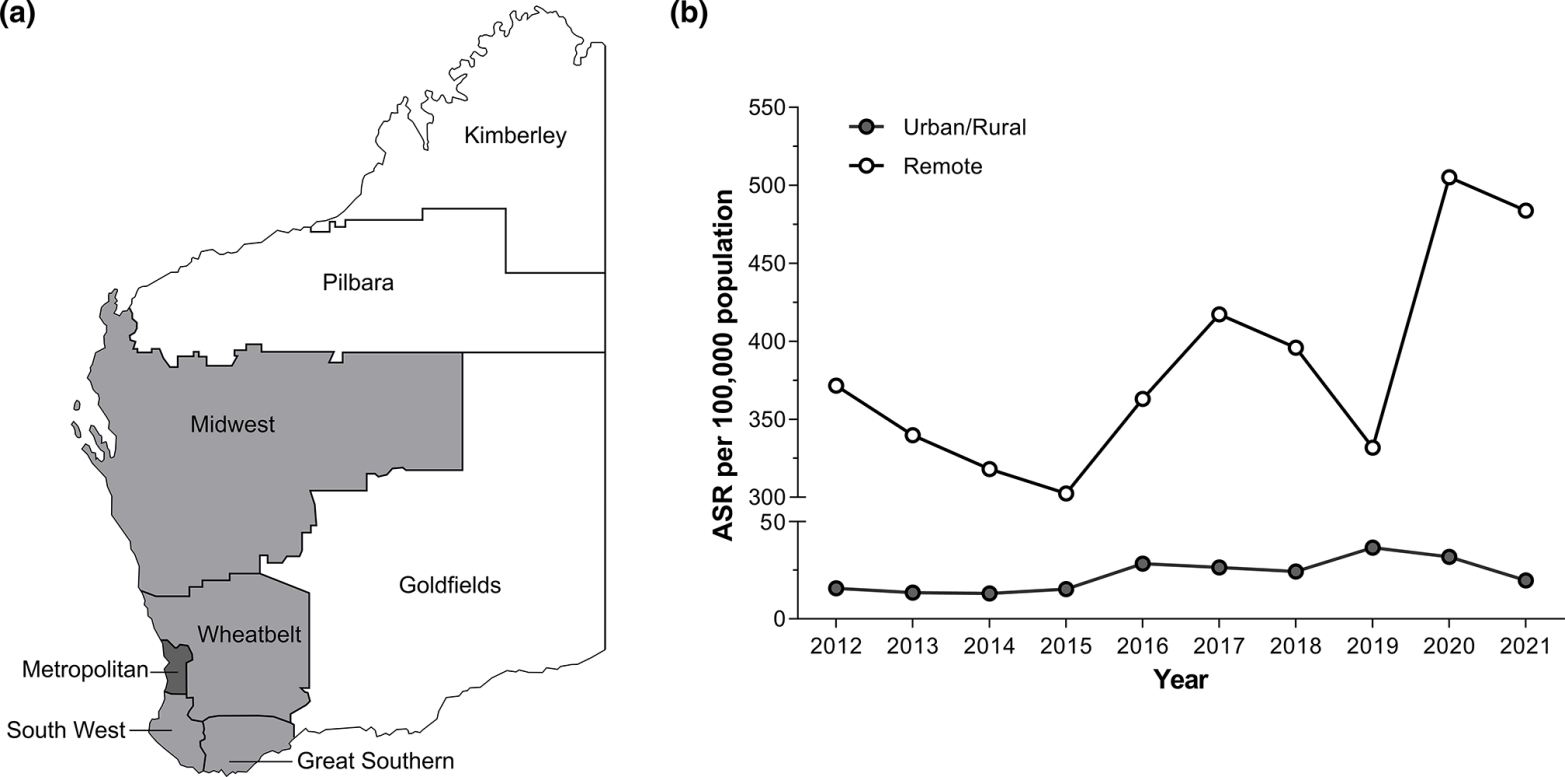
Western Australian health regions (**a**) and notification rates by region (**b**). (**a**) Metropolitan Perth, the Southwest, Great Southern and Wheatbelt (grey) grouped together as urban/rural regions and the Kimberley, Pilbara, Midwest and Goldfields (white) grouped together as remote regions for surveillance proposes. (**b**) Gonorrhoea notification rate per 100 000 population by Western Australian health regions from 2015 to 2021. Data were extracted from the literature [11] and online (https://ww2.health.wa.gov.au/Articles/N_R/Notifiable-infectious-disease-report?report=gonorrhoea).

Between 2013 and 2017, the notification rate of gonorrhoea in Australia increased by 80 % from 65.5 to 118/100 000 population [10]. In WA, which represents 11 % of the total population, rates increased by 63 % from 77 to 125/100 000 notifications over the same period. For infectious disease notification purposes, WA is divided into eight geographical regions: the Kimberley, Pilbara, Midwest and Goldfields regions are considered remote, the Great Southern, South West and Wheatbelt regions are considered rural, and the Perth metropolitan area is considered urban. Collectively, 97 % of WA’s population live in the urban/rural regions (Fig. 1). In 2017 gonorrhoea notification rates in WA’s three remote regions were between three- and ten-fold higher than that of urban/rural regions [11]. Approximately 30 % of isolates in the urban/rural regions exhibited a multi-drug-resistant (MDR) phenotype, defined as resistance to more than two antimicrobial classes [12]. A small prospective study of the genomic epidemiology of WA gonococcal isolates from 2011 to 2013 revealed a predominance of international gonococcal lineages in urban/rural regions whilst the remote regions were dominated by two genomic population groups, Aus1 and Aus2 [9].

Ongoing antimicrobial surveillance in WA indicates the majority of gonococcal isolates from the remote regions remain susceptible to penicillin. Therefore, WA is unique in that two treatment regimes are used dependent upon the geographical region: cases in urban/metropolitan areas are treated with ceftriaxone (Cef) while penicillin is still utilized in remote settings. In 2018, the first two cases of gonorrhoea caused by extensively drug-resistant (XDR) isolates with dual resistance to Cef and azithromycin (Azi) were reported in Australia [13]. The two cases had travel links to Southeast Asia and the isolates were identified as *N. gonorrhoeae* A2543, a strain previously reported in the UK (WHO Q) [14, 15] that was also associated with travel to that region. Clonal spread of low-level Azi-resistant (AziLR) *N. gonorrhoeae* has also been reported in Australia, including an outbreak in MSM in New South Wales (NSW). The AziLR strain harboured the meningococcal-type *mtrR*-encoded efflux pump regulator which is associated with reduced susceptibility/low-level resistance to Azi [14, 16]. After the NSW outbreak, there was an outbreak of an AziLR penicillinase-producing *N. gonorrhoeae* (PPNG) strain in the heterosexual population in South Australia (SA) [17].

Ongoing surviellence of the provenance of gonorrhoea in WA serves as an early warning system for bridging across these communities and alerts the public health system to changes in treatment recommendations. To understand the provenance of isolates causing gonorrhoea in WA, we have performed a genomic epidemiology survey of isolates collected during 2017. We utilized iPLEX testing as a low-resolution means of parsing the collection for the main genetic clusters and then used whole genome sequencing (WGS) to examine antimicrobial resistance traits and genocoding across multiple geographical regions and links to international travel.

## Methods

### Collection and culture of *N. gonorrhoeae* isolates

All private and public pathology laboratories refer gonococcal isolates to the Western Australian Gonococcal Surveillance Programme (WAGSP) for standardized antimicrobial sensitivity (AMS) testing. In 2017, 3 341 notifications were confirmed by PCR and 22 % resulted in cultured isolates. Penicillin, ciprofloxacin, spectinomycin, azithromycin, ceftriaxone and tetracycline susceptibility tests were performed by agar dilution to produce antibiograms as previously described [18]. Isolates with a ceftriaxone minimum inhibitory concentration (MIC) ≥0.06 mg l^−1^ or an azithromycin MIC ≥1 mg l^−1^ were retested by an E-test as per the manufacturer’s guidelines [19, 20]. Table S1 (available with the online version of this article) is an interpretive criterion table for MIC values for *N. gonorrhoeae *.

Isolates from the same patient collected from different anatomical sites or within 3 months with the same antibiogram profile were excluded from the study.

### Characterization of gonococcal genotypes by the iPLEX genotyping system

The gonococcal isolates were genotyped by the iPLEX genotyping system, a previously described high-throughput informative SNP typing system using Agena Bioscience iPLEX genotyping technology [21]. The system includes the iPLEX multilocus sequence type (MLST) method which targets 14 informative SNPs to predict the MLST [22] and the iPLEX antimicrobial resistance (AMR) method which targets 11 chromosomal AMR mutations located on the gonococcal 23S rRNA, *gyrA*, *mtrR*, *penA* and *ponA* genes [8]. The SNP profiles obtained from the iPLEX-MLST and iPLEX-AMR are combined to determine a unique genotype (WA-1 to WA-78). Due to the limited number of SNPs used by iPLEX typing for MLST, all possible MLST-STs associated with each WA-type are listed in Table S2.

### Whole genome sequencing and phylogenetic analysis

Genomic DNA extraction was performed using the DNeasy Blood and Tissue Kit (Qiagen) as per the manufacturer’s instructions and used as input in the Nextera XT library preparation protocol. WGS was performed on the Illumina NextSeq 500 platform (Illumina) using 150 bp paired-end chemistry. Sequence reads were assembled using SPAdes version 9.0 [23]. The quality of the assembled genomes was assessed using the Quast genome assembly evaluation tool [24]. Bacterial Isolates Genome Sequence database (BIGSdb) genomics platform tools hosted on https://pubmlst.org/neisseria/ were used for annotation and genome-wide analysis of the assembled genomes [25]. All genomes have been provided with a PubMLST ID (Table S3). The core genome analysis was performed on the 196 *N *. *gonorrhoeae* isolates included in this study [126 *N *. *gonorrhoeae* isolates from this study, 11 PPNG/AziR strains from Queensland (Qld) isolated in 2017 (unpublished, Dr David Whiley) and 59 isolates collected in 2011–2013 [9]]. The core genome was determined using the genome comparator tool at BIGSdb using the *N. gonorrhoeae* cgMLST v1.0 scheme set to the default settings of a core threshold of 100 % (10 December 2019). This process revealed that of the 1647 defined loci in the database, 970 were present in 100 % of the 196 isolates (the list of loci is given in Table S4). The SNP-sites command-line tool was used to extract SNPs from the core genome multi-FASTA alignment [26]. mega 7 software was used to create a maximum-likelihood core genome SNP phylogenetic tree using previously described settings [9, 27]. FastGEAR software, which is based on the Bayesian Analysis of Population Structure (BAPS) clustering algorithm [28], was used to reveal genomic population structure and to analyse recombination patterns between different genomic structure groups [29].

All whole genome sequences from this study will be available in PubMLST (for PubMLST IDs see Table S3). All raw sequences associated with this publication have been submitted to the Bioproject PRJNA868503: *Neisseria gonorrhoeae* raw sequence reads (TaxID: 485) at NCBI.

### Genotyping and identification of AMR determinants from WGS

*N. gonorrhoeae* multi-antigen sequence typing (NG-MAST [30]) and *N. gonorrhoeae* sequence typing for antimicrobial resistance (NG-STAR [31]) typing were performed using a genome comparator tool (http://pubmlst.org/neisseria/). The sequences were submitted to the NG-MAST (http://ng-mast.net/) and NG-STAR (https://ngstar.canada.ca/) databases for sequence type (ST) determination. All novel alleles and allelic combinations were referred to each database curator to be assigned a new allelic number and ST, respectively.

Through single-linkage clustering, isolates were clustered into core genome groups using a threshold of 400 or fewer locus differences [32] which was performed using *N. gonorrhoeae* cgMLST v1.0 on 25 November 2022. The core genome clusters (Ng_cgc_400) for each sequenced isolate are shown in Table S3. The relationship of Ng_cgc_400 with WA-types is shown in Fig. S1.

The genome comparator tool at PubMLST [33] was used to detect and identify mutations in AMR loci (*ponA*, *penA*, *porB*, *gyrA*, *parC*, *mtrR*, ^pro^ *mtrR*, *bla*-TEM and *tetM*) using the *N. gonorrhoeae* AMR scheme. *N. gonorrhoeae* has four copies of the 23S rRNA gene and the command line program SRST2 was used to identify mutations in 23S rRNA directly from WGS Illumina raw sequence reads [34]. The number of mutated 23S rRNA copies was calculated from the SRST2 pileup output files as the depth of mutated reads compared to the depth of wild-type (wt) reads. If 100 % of k-mers mutated the four alleles were called mutated, if 75 % of k-mers mutated 3/4 alleles were called mutated, 50 % for 2/4 mutated alleles, 25 % for 1/4 mutated alleles, and 0 % mutated k-mers were called wt. The conjugative and β-lactamase plasmids and the gonococcal genetic islands (GGIs) were also detected using the genome comparator tool at the PubMLST.

The GrapeTree genetic relationships visualization tool [35] on the PubMLST website [33] was used to generate a minimal spanning tree of a publicly available collection of 4 656 *N *. *gonorrhoeae* isolates [25]. The tree was generated using an *N. gonorrhoeae* cgMLST scheme of 1 649 loci and coloured by country of isolation.

### Statistical analysis

RStudio and Epi Info software packages were used for descriptive statistical analyses of the data and to generate figures and maps [36, 37].

## Results

### Demographics and geographical distribution of the cultured gonococcal isolates collected in 2017

Unique isolates were selected from 510 male (median age 30 years) and 231 female (median age 25 years) (2.2 : 1 male to female ratio). Overall, 623 (84.1 %) and 118 (15.9 %) of the 741 isolates were from urban/rural and remote regions respectively. Isolates from urban/rural patients were collected from multiple sites including the uro-genital tract (81 %), rectum (9.3 %), throat (6.7 %) and eyes (0.4 %). Twelve isolates (1.6 %) were from disseminated gonococcal infections (DGIs). Apart from three DGIs, all isolates referred from the remote regions were collected from uro-genital sites.

### AMR profiles from antibiograms

Fifteen antibiogram profiles were identified from the 741 gonorrhoea isolates (Table 1). The most frequent profile, penicillin less-susceptible (PLS) (MIC 0.06–0.5 mg l^−1^) with no antimicrobial co-resistances (64 %, *n*=475/741), was identified in 60 % of the urban/rural region isolates (373/623) and 86 % (*n*=112/118) of remote region isolates. The PPNG/ciprofloxacin resistant (CipR) (MIC ≥1 mg l^−1^) profile was identified in 11 % (*n*=83) of isolates, which accounted for 12.5 % (*n*=78) of the urban/rural isolates and 4 % (*n*=5) of the remote isolates. Only 8.5 % (*n*=63) of isolates were penicillin highly susceptible (PHS) (MIC ≤0.03 mg l^−1^) with no antimicrobial co-resistances, which accounted for 9 % (*n*=56) of the urban/rural isolates and 6 % (*n*=7) of the remote isolates. AziLR (MIC ≥1 mg l^−1^) PHS isolates accounted for 3.9 % (*n*=29) of isolates from the urban/rural regions. Ten PLS, four PPNG, and one CipR and PLS were also AziLR. Only three of the 44 AziLR isolates were from the remote regions. High-level azithromycin resistance (MIC ≥256 mg l^−1^) was not detected. All nine isolates with decreased susceptibility to ceftriaxone (Cef DS) (MIC ≥0.06 mg l^−1^) were from the urban/rural regions and were co-resistant to ciprofloxacin, two-thirds (6/9) had chromosomally mediated resistance to penicillin (CMRP) and none were resistant to azithromycin.

**Table 1.** Susceptibility profiles of *N. gonorrhoeae* by WA region

Penicillin resistance*	Ceftriaxone resistance	Ciprofloxacin resistance	Azithromycin resistance	Percentage urban/rural (*n*)	Percentage remote (*n*)	Percentage total (*n*)
PLS	S	S	S	79 % (373)	21 % (102)	64 % (475)
PPNG	S	R	S	94 % (78)	6 % (5)	11 % (83)
PHS	S	S	S	89 % (56)	11 % (7)	9 % (63)
PHS	S	S	R	100 % (29)	0 % (0)	4 % (29)
CMRP	S	S	S	100 % (18)	0 % (0)	2 % (18)
PLS	S	R	S	93 % (14)	7 % (1)	2 % (15)
PPNG	S	LS	S	100 % (13)	0 % (0)	2 % (13)
PLS	S	R	R	100 % (10)	0 % (0)	1 % (10)
PLS	S	S	R	100 % (9)	0 % (0)	1 % (9)
CMRP	DS	R	S	100 % (6)	0 % (0)	1 % (6)
PLS	S	LS	S	100 % (5)	0 % (0)	1 % (5)
PPNG	S	S	R	25 % (1)	75 % (3)	1 % (4)
CMRP	S	R	S	100 % (4)	0 % (0)	1 % (4)
PPNG	S	S	S	100 % (4)	0 % (0)	1 % (4)
PLS	DS	R	S	100 % (3)	0 % (0)	0 % (3)

*PPNG=penicillinase-producing *N. gonorrhoeae*; CMRP=chromosomally mediated resistance to penicillin; PHS=penicillin highly susceptible (MIC ≤0.03 mg l^−1^); PLS=penicillin less susceptible (MIC 0.06–0.5 mg l^−1^); DS=ceftriaxone decreased susceptibility (MIC ≥0.06 mg l^−1^); LS=ciprofloxacin low susceptibility (MIC 0.06–0.5 mg l^-1^). S=susceptible; R=resistant.

### Geographical distribution by iPLEX genotype

Genotyping was successfully performed on 99 % (*n*=734/741) of the isolates as 1 % grew poorly on GCA agar. Seventy-eight iPLEX genotypes were identified (WA-1 to WA-78) (Table S2). Twenty predominant genotypes (defined as having more than five isolates) accounted for 88 % (*n*=645/734) of the isolates. Four genotypes accounted for almost half of the isolates: WA-10 (164 isolates, 22 % of isolates); WA-14 (74, 10 %); WA-24 (56, 8 %); and WA-29 (56, 8 %).

**Fig. 2.**
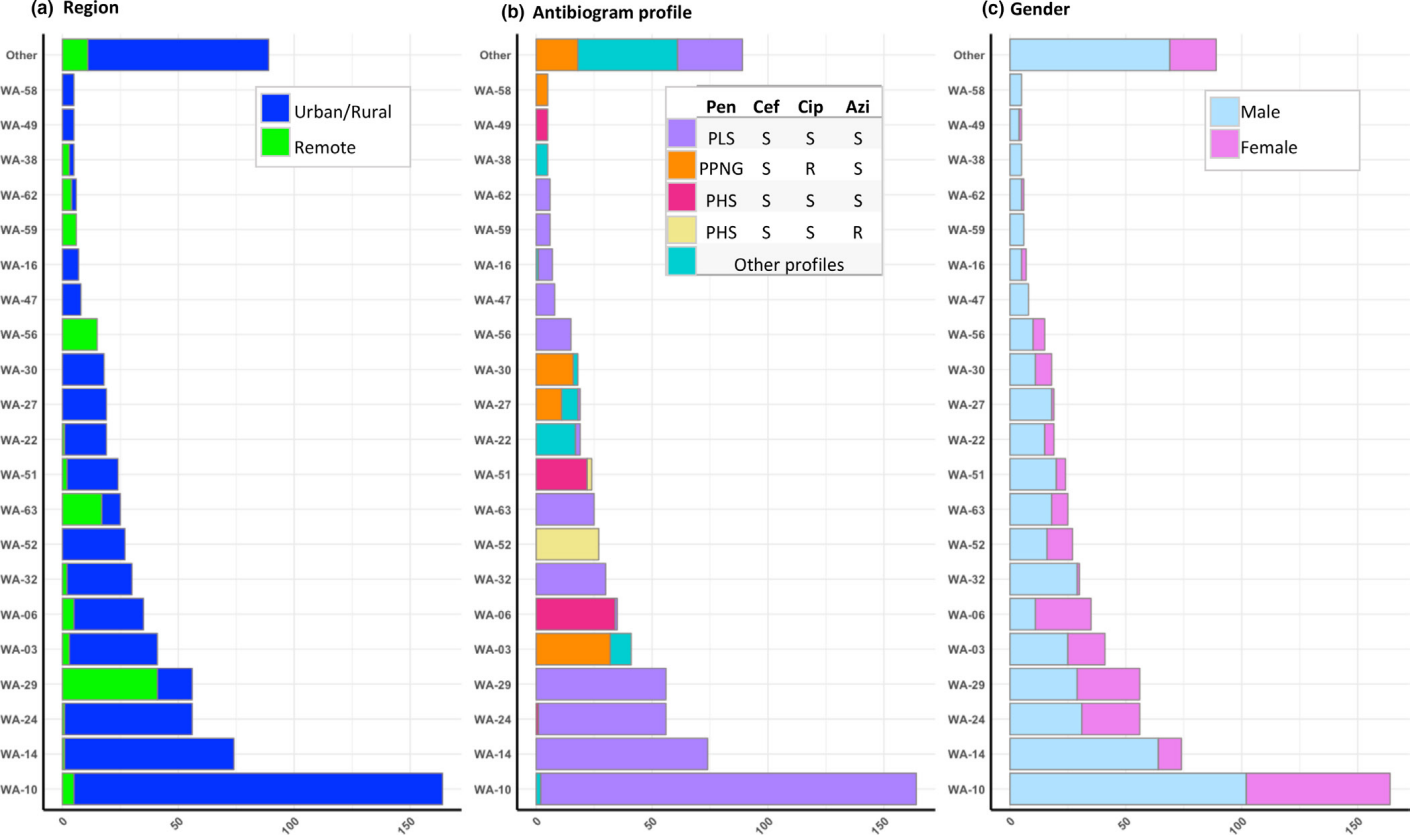
Bar charts showing the frequency of the most common WA iPLEX genotypes coloured by region (**a**), antibiotic resistance profile (**b**) and gender (**c**). Bars are coloured based on the proportion of isolates showing a specific antibiotic profile. Penicillin (Pen), ceftriaxone (Cef), ciprofloxacin (Cip) and azithromycin (Azi). Penicillinase-producing *N. gonorrhoeae* (PPNG), penicillin less susceptible (PLS), penicillin highly susceptible (PHS), susceptible (S) and resistant (R).

There was an association between the iPLEX genotype and geographical region (Fig. 2a). Five of the genotypes, WA-29, WA-56, WA-59, WA-62 and WA-63, accounted for 91 % (108/118) of isolates from the remote regions. The five predominant remote region genotypes were also isolated from a small proportion of patients living in the urban/rural regions, indicating that these genotypes had travelled from remote to urban/rural regions. In contrast, Perth metropolitan predominant genotypes, WA-10 and WA-14, were not identified in the remote regions, indicating little/no travel of genotypes from urban/rural to remote areas.

Nine of the 20 predominant genotypes were PLS with no co-resistances: urban/rural region genotypes WA-10, WA-14, WA-24 and WA-32, and the remote region genotypes WA-29, WA-6, WA-56, WA-59 and WA-62 (Fig. 2b). WA-14 and WA-32 isolates were primarily from male patients living in the urban/rural regions (Fig. 2c). All PLS phenotype isolates harboured a *penA* allele with the 345D insertion.

Predominant genotypes WA-3, WA-27 and WA-30 were PPNG/CipR. Ciprofloxacin resistance in the 83 PPNG/CipR isolates was due to GyrA S91F and D95G/A mutations.

The nine Cef DS isolates(MIC 0.06–0.125 mg l^−1^) were only detected in the Perth metropolitan area and consisted of four genotypes: WA-21, WA-40, WA-41 and WA-77. The mosaic *penA* allele was found in two Cef DS WA-21 isolates.

Isolates with the PHS phenotype were primarily identified in four predominant genotypes, WA-6, WA-51, WA-52 and WA-49, and all 92 PHS isolates harboured a wild-type (WT) *penA* allele with no 345A insertion. WA-51 and WA-52 also harboured the AziLR determinant. In addition, WA-52 possessed a C2611T mutation in 23S rRNA. All WA-51 isolates carried at least one copy of the WT and C2611T mutation in 23S rRNA loci.

Low azithromycin resistance was present in multiple genotypes, including the five PPNG/AziLR WA-38 isolates, of which three were from remote regions. Four AziLR isolates belonging to genotype WA-13 were characterized by a meningococcal-type *mtrR* variant that confers resistance to azithromycin [14]. Low azithromycin resistance was associated with other non-predominant genotypes, including two isolates of WA-12 and one each of WA-9 and WA-15.

### Relationship of local genomic epidemiology to the international genomic epidemiology using the cgMLST scheme

Due to the limited number of SNPs used by iPLEX typing, it is not possible to get the exact MLST match to establish the global context of the isolate collection. To investigate genomic epidemiology of the WA isolates within the international context, isolates representing each iPLEX genotype were whole genome sequenced which confirmed the MLST type with 96 % congruency between the two methods (Table S2). In those instances where the methods disagreed, only one isolate was whole genome sequenced and was missing the necessary sequence data for a housekeeping gene (data not shown). There was reasonable congruence of the WA-genotype with the established typing scheme NG-STAR, which uses seven alleles associated with AMR [31]. NG-MAST [30], which examines variability within the hypervariable sections of the outer membrane antigens, *porB* and *tbpB*, showed that there was considerable heterogeneity between isolates in each MLST cluster. To understand the total genetic diversity in the collection, a minimal spanning tree was generated using the cgMLST scheme using Ng_cgc_400 [32], which clusters 400 or fewer locus differences, thus accounting for all variable loci in the dataset. This analysis revealed 21 clusters of related locus differences (Fig. S1, Panel A). When this cgMLST tree was overlaid with WA-type, it revealed that WA-56 and WA-63 were more clonal than the other WA-genotypes, which were genetically diverse (Fig. S1, Panel B).

**Fig. 3.**
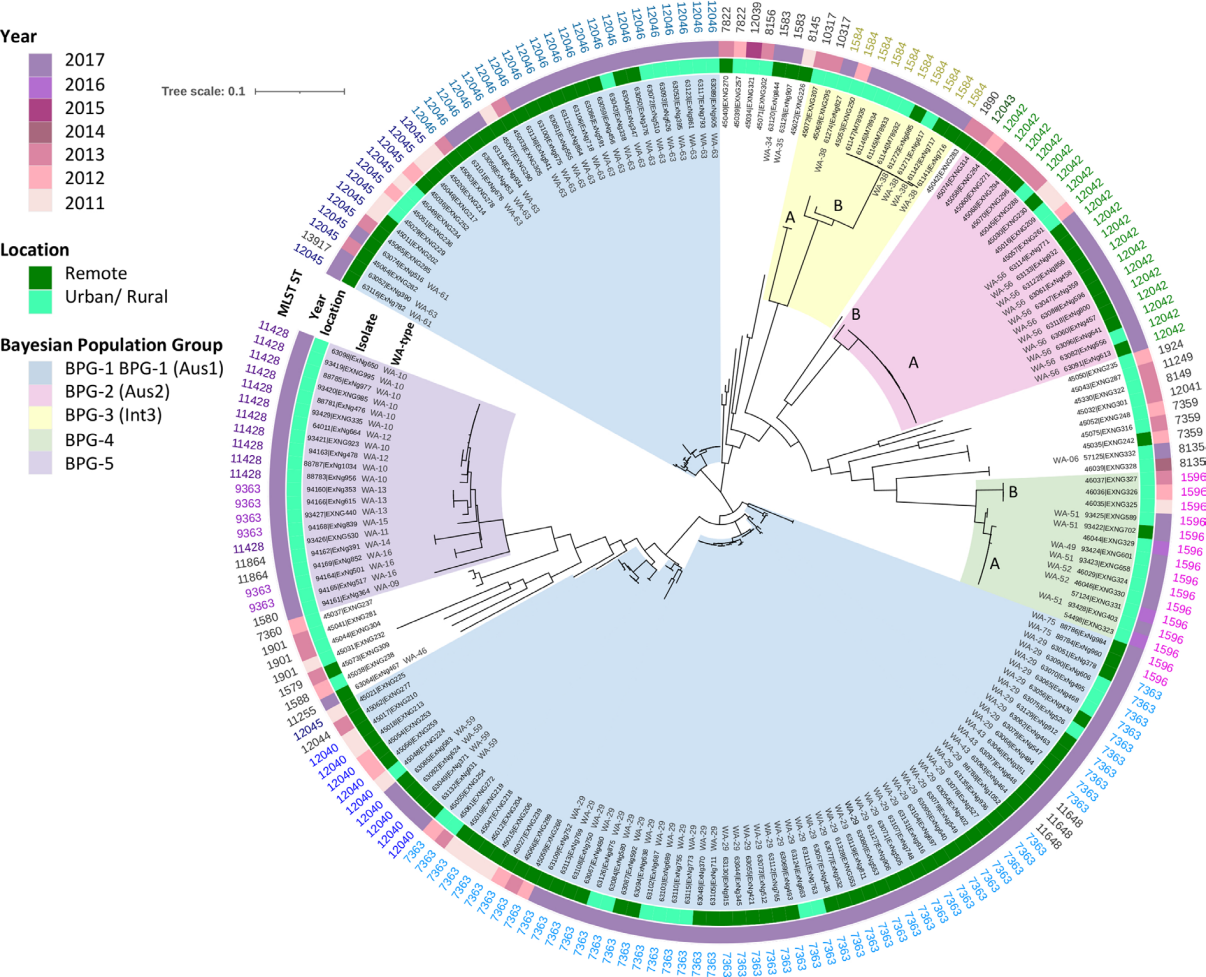
Core genome maximum likelihood phylogeny of 196 *N *. *gonorrhoeae* isolates collected from 2011 to 2017 in Australia. Inner to outer rings are: WA-type, isolate identifier, year of collection, geographical region and MLST sequence types. Collection includes WA isolates (59 isolates from 2011 to 2013 [9] and 133 isolates from this study) and four isolates from Queensland (see Methods). Bayesian population groups (BPGs 1–5) are coloured in radial sections according to the key. The phylogeny was generated with 100 bootstrapped replicates based on cgMLST SNPs. Distinct phylogeny clusters of BPGs 2 and 3 and were annotated with letters A and B.

To understand the evolutionary relationships further, a core genome SNP phylogeny and genomic population structure analysis were performed on 133 isolates representing WA- iPLEX genotypes with more than five isolates and all isolates with an AMR phenotype (Fig. 3). In addition, 59 isolates from a previous collection between 2011 and 2013 from WA [9] and four Queensland isolates were included (see Methods). Population structure analysis using FastGEAR based on BAPS revealed six main Bayesian population genogroups (termed BPG-1 to BPG-6) which remained stable when accounting for ancestral recombination in the dataset (Fig. S2). BPG-1 and BPG-2 were associated with remote regions, while BPG-3, BPG-4, and BPG-5 were associated with urban/rural regions of WA. BPG-1 and BPG-2, and BPG-3 corresponded to the previously identified Aus1 and Aus2, and international group Int3, respectively, in a retrospective study of 59 isolates collected in WA between 2011 and 2013 [9]. BPG-6 contained the most genetically divergent isolates, which do not represent a biologically meaningful cluster (Fig. 3).

To investigate evolutionary patterns of variation between different BPGs, the FastGEAR analysis tool was run on the core genome alignment using default settings to detect both recent and ancestral recombination events that occurred after or before divergence of the BPGs, respectively (Fig. S2). This analysis showed all BPGs are affected by minimal recent recombination events, with BPG-2 showing the lowest degree of recombination. The recent recombination events were mutual between BPG-1 to BPG-2. BPG-3, BPG-4 and BPG-5 populations showed higher diversity of origin of imported genomic fragments. BPG-2 is predicted to have a high degree of ancestral recombination mostly with BPG-1, BPG-3 and BPG-4. The BPG-5 population also showed a high rate of ancestral recombination but with multiple lineages. Unlike other populations, BPG-1 was estimated to have no ancestral recombination events (Fig. S2).

Each BPG consistently represented a single or multiple closely related iPLEX-genogroups (Fig. 3). BPG-1 corresponded to Aus1 and encompassed iPLEX-types WA-29, WA-63, WA-43, WA-59, WA-61 and WA-75. In BPG-1, iPLEX-genogroups mostly corresponded to a single MLST ST, but there were exceptions. For example, although WA-63 mostly corresponded to ST12046 (*n*=19/20), one isolate (EXNG390) was designated an ST13917 which has arisen due to one SNP (G378C in *pgm* locus). The allelic difference between MLST ST12046 and ST13917 could be a result of a recombination event as the *pgm* allele of ST13917 is identical to the closely related MLST ST12045 (*pgm* allele 223). EXNG390 had more than 300 SNP differences in the core genome compared to the other 19 isolates of ST12046. BPG-2 contained all Aus2 isolates, which were all WA-56. There were two subgroups, BPG-2A and BPG-2B. BPG-2A isolates (*n*=20), all with MLST ST12042, had fewer than 20 SNP differences in their core genome. BPG-2B was a single outlier strain, EXNG314 (MLST ST12043), and had over 280 SNP differences from BPG-2A. BPG-3 was represented by MLST ST1584 and ST10317 but was iPLEX-MLST typed as WA-38. ST1584 was represented by a central clonal subgroup A (*n*=8/10) with fewer than 25 SNP differences in their core genome. Two of the subgroup A isolates, EXNG827 and EXNG250, were outliers, having more than 1 000 SNP differences from the main subgroup A. BPG-4 consisted of MLST ST1596, which corresponded to iPLEX types WA-49, WA-51 and WA-52. BPG-4B, which accounted for 61 % of BPG-4 isolates (*n*=8/13), was clonal, with fewer than 40 SNP differences in the core genome of this branch. BPG-4A was represented by three clonal isolates which were conserved with fewer than five SNP differences between them, but over 360 SNP differences distinguished the isolates from BPG-4B isolates. BPG-5 was the most genetically diverse group in terms of iPLEX genotypes as it contained seven WA-types (WA-9 to WA-16) distributed amongst four different MLST STs (ST11428, ST9363, ST11864 and ST1580).

**Fig. 4.**
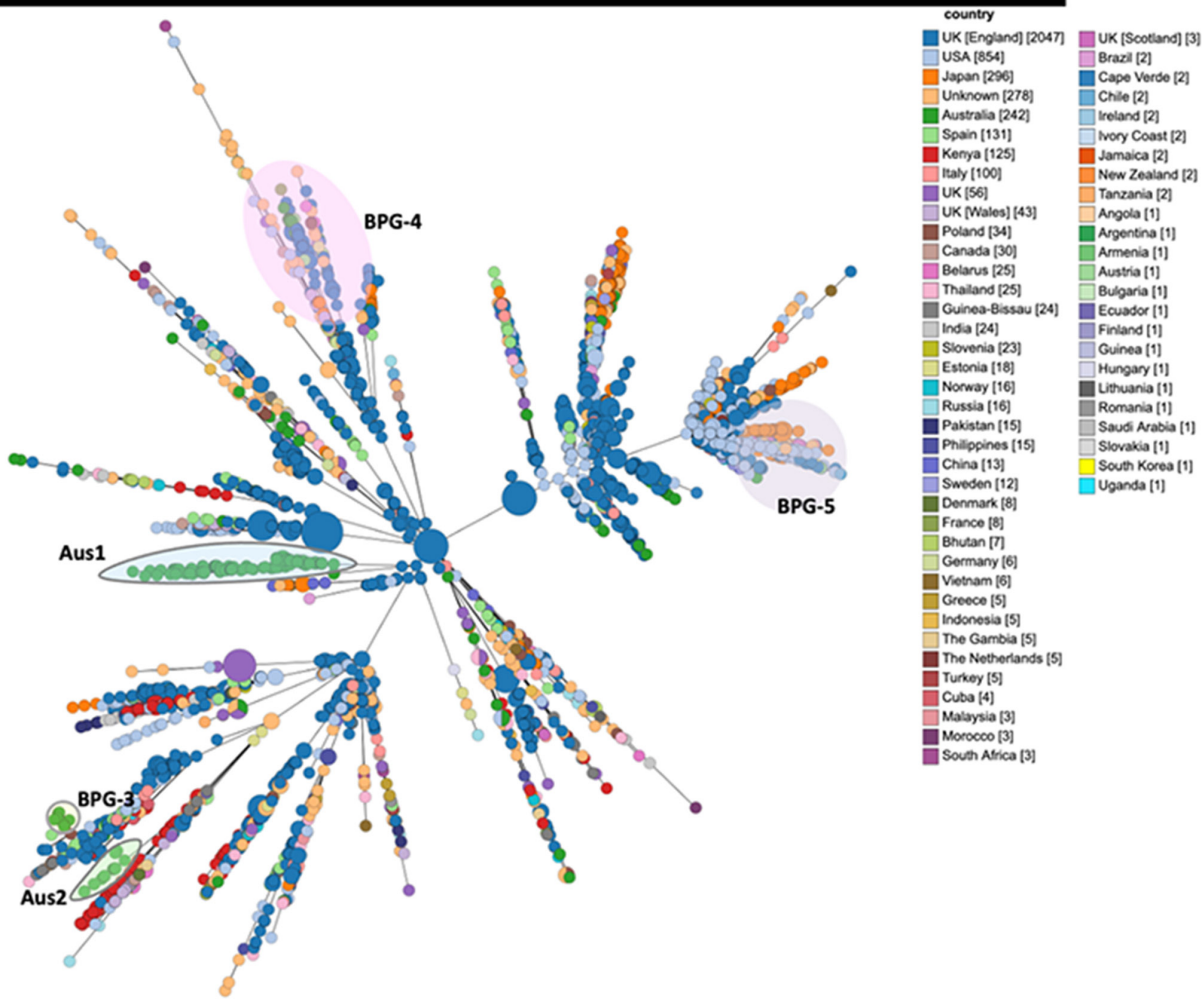
Genetic relationship of BPG-1 to BPG-5 to all isolates in the PubMLST database (January 2019). Minimum-spanning tree of 4 656 *N. gonorrhoeae* isolates based on allelic profiles generated from the core genome MLST of 1 649 genes determined by the GrapeTree analysis tool implemented in the PubMLST database [25, 35]. Nodes are coloured by country, and size of the node is directly proportional to the number of isolates. The BPGs are circled – the solid outline of groups includes only the Australian isolates. They are labelled as Aus1 (BPG-1, 105 isolates), Aus2 (BPG-2, 20 isolates), BPG-3 (12 isolates), BPG-4 (13 isolates) and BPG-5 (20 isolates).

We hypothesized that the diversity of WA-types in BPG-4 and BPG-5 may indicate the isolates in the two groups are of international origin and have been introduced over time into WA. To examine this further, a minimal spanning tree of all 4 656 *N *. *gonorrhoeae* isolates publicly available in the PubMLST isolate database was constructed based on the allelic profiles of the cgMLST scheme using the GrapeTree tool and were coloured by country of isolation. Aus1 was unique to the Australian context, having been collected from Qld (Fig. 4) as well as WA, while Aus2 was only isolated in WA. Although BPG-3 formed a distinct cluster and representatives have only been collected from Australia, it is closely related to a larger group of isolates mostly collected from the UK. BPG-4 and BPG-5 isolates have been collected from multiple international regions, indicating many isolates that have spread globally over the last 59 years (1960–2019) (Fig. 4).

### Association of Cef DS and AziR with inter-state and international genetic lineages

The nine Cef DS isolates (genotypes WA21, WA-40, WA-41 and WA-77) appeared to be sporadic introductions and were unrelated to the BPG-groups. WA-21 corresponded to ST1901, an international lineage carrying a mosaic *penA*, which was detected by iPLEX typing. WA-41 and WA-42 correlated with ST7827, but all carried a wild-type *penA* allele.

**Fig. 5.**
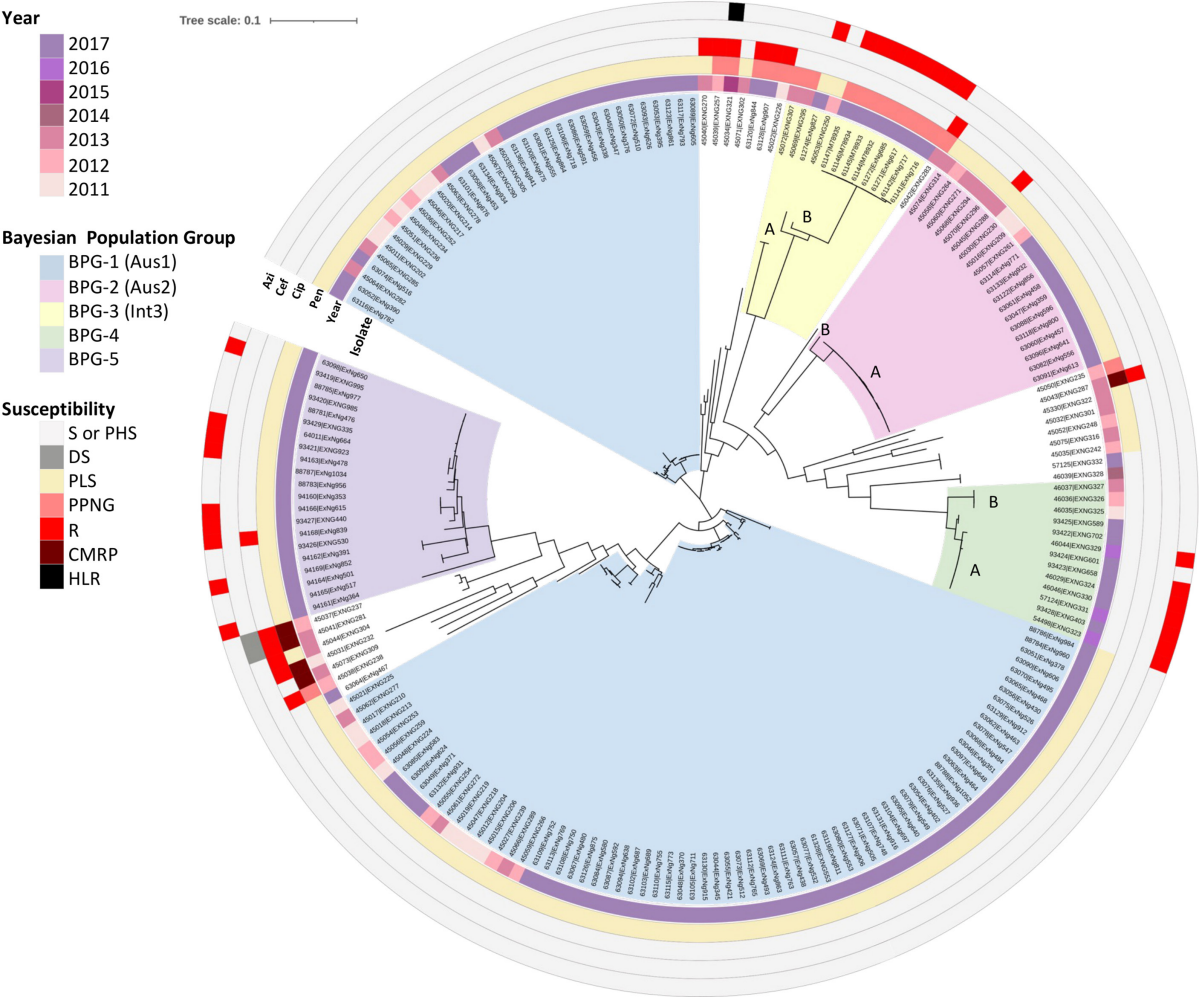
Core genome maximum likelihood phylogeny of 196 *N *. *gonorrhoeae* isolates collected from 2011 to 2017 showing antibiotic profiles. Inner to outer rings are: isolate identifier, year of collection, and susceptibility to penicillin (Pen), ciprofloxacin (Cip), ceftriaxone (Cef) and azithromycin (Azi). Strain collection includes 59 WA isolates from 2011 to 2013 [9] and 133 isolates from this study and four isolates from Queensland (see Methods). Key to antibiogram definitions: susceptible (S), penicillin highly susceptible (PHS), decreased susceptibility (DS), penicillin less susceptible (PLS), penicillinase producing *N. gonorrhoeae* (PPNG), resistant (R) and chromosomally mediated resistance to penicillin (CMRP). Bayesian population groups (BPGs 1 to 5) are coloured in radial sections according to the key. The phylogeny was generated with 100 bootstrapped replicates based on cgMLST SNPs.

AziR isolates belonged to BPG-3, BPG-4 and BPG-5 (Fig. 5). The internal population structures of BPG-3 and BPG-4 supported a model for a clonal introduction and local expansion in urban/rural WA [5]. BPG-5 comprised isolates collected in 2017 from urban/rural WA, suggesting a recent introduction of either inter-state or international *N. gonorrhoeae* strains into urban WA.

**Fig. 6.**
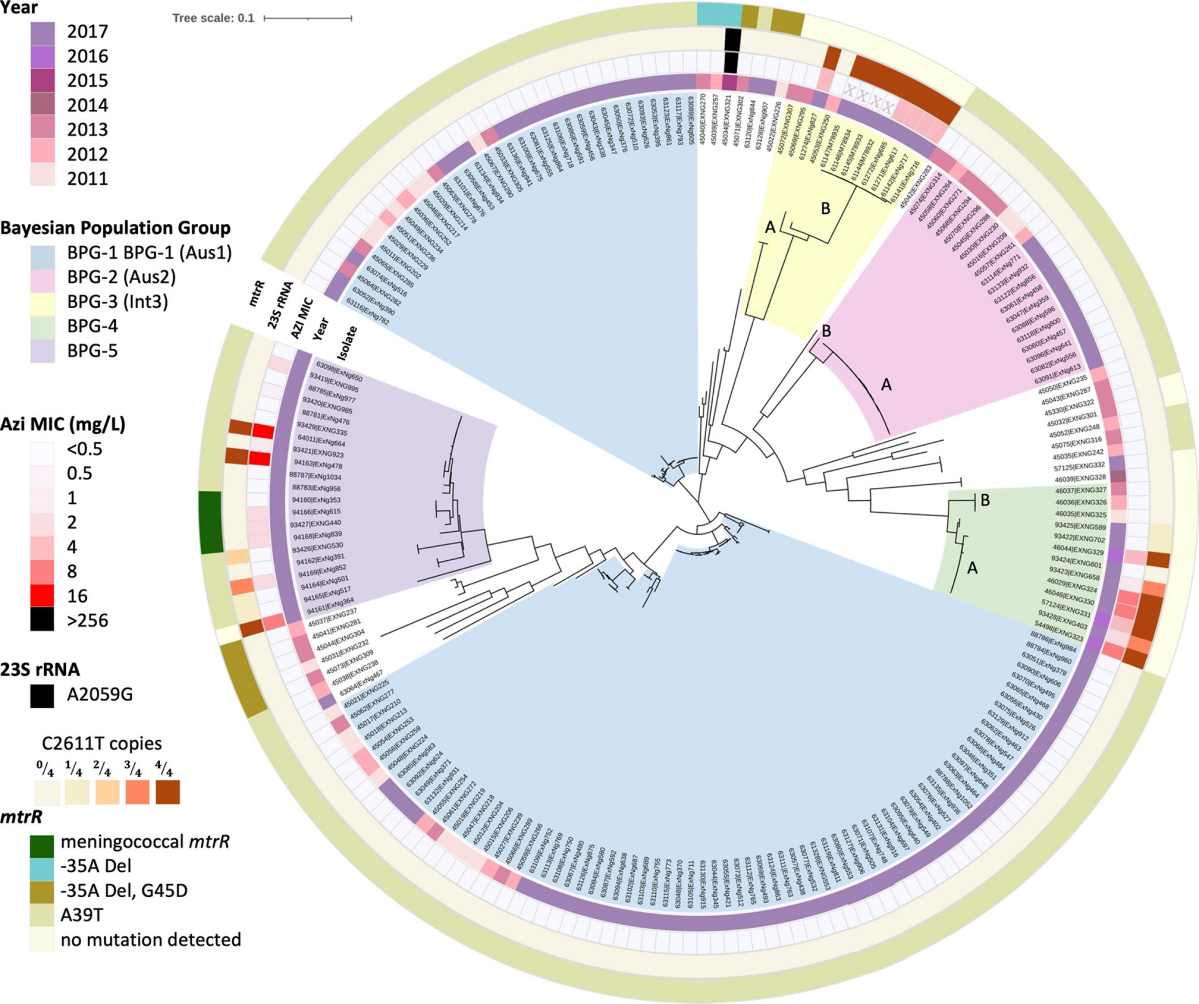
Core genome maximum likelihood phylogeny of 196 *N *. *gonorrhoeae* isolates collected from 2011 to 2017 in Australia, showing azithromycin minimum inhibitory concentration (Azi MIC) and azithromycin-resistant determinants (23S rRNA mutations and *mtrR* mutations). Inner to outer rings are: isolate identifier, year of collection, azithromycin MIC, 23S rRNA mutation and *mtrR* promoter mutation. Strain collection includes 59 WA isolates from 2011 to 2013 [9] and 133 isolates from this study and four isolates from Queensland (see Methods). Bayesian population (BAPs) groups are coloured in radial sections according to the key. The phylogeny was generated with 100 bootstrapped replicates based on cgMLST SNPs.

Whilst all BPG-3 isolates from the 2011–2013 dataset were azithromycin-susceptible, the five BPG-3 isolates collected in 2017 were AziLR. BPG-3A consisted of isolates collected in 2013 and were PPNG/AziS, suggesting the group could have been an ancestral population from which BPG-3B evolved and acquired AziLR. Three isolates (EXNG685, EXNG716 and EXNG717) were obtained from a remote region in WA and clustered with the PPNG/AziLR isolates from Qld (see Methods), suggesting that there may have been a common travel and community history. All AziLR strains in BPG-3 contained the 23S rRNA C2611T mutation that confers low-level resistance to azithromycin (Fig. 6). BPG-4 subgroups BPG-4A and BPG-4B had a similar history. BPG-4B isolates were PHS/AziS, genetically diverse and were isolated in urban/rural regions prior to 2017. BPG-4A isolates were collected in urban/rural WA in 2017, were clonal and had acquired AziR. The observation that all BPG-4B isolates carried WT 23S rRNA copies and that BPG-4A isolates showed varying frequencies of C2611T mutated 23S rRNA copies (zero to four mutated copies) supports the development of azithromycin resistance in BPG-4A (Fig. 6).

BPG-5 was the most genetically diverse group, consisting of 21 isolates collected in 2017 from urban/rural WA. All isolates showed mutations in *mtrR* associated with azithromycin resistance, either the A39T substitution (81 %, *n*=17/21) or the meningococcal *mtrR* allele (19 %, 4/21). In total, 48 % (*n*=10/21) of BPG-5 isolates had varying levels of azithromycin reduced susceptibility/resistance phenotypes (MIC 0.5–16 mg l^−1^), of which four isolates displayed the C2611T mutation in 23S rRNA copies (Fig. 6). The portion of C2611T mutated 23S rRNA copies correlated with the azithromycin MICs obtained. Isolates with azithromycin MICs (4 and 16 mg l^−1^) had all 23S rRNA copies mutated. Isolates with azithromycin MICs (1–2 mg l^−1^) had three of the four 23S rRNA copies mutated and isolates with MICs≤0.5 mg l^−1^ had fewer than three copies mutated. Two of the WA-10 AziR isolates did not carry the C2611T mutation or the *mtrR* allele (EXNG923 and EXNG995). These isolates possessed allele 1006 of MtrD (NEIS1633) in which there has been a K823E amino acid change associated with improved efflux of azithromycin [38].

## Discussion

Our study investigated the genetic diversity and antibiotic resistance profiles of *N. gonorrhoeae* isolated in WA in 2017 and compared them to international lineages. Genotyping of 734 of the isolates using the iPLEX assay identified 78 genotypes, of which approximately 50 % belonged to four genotypes (WA10, WA-14, WA-24 and WA-29). Overall, 37 % (*n*=29/78) of genotypes had previously been identified in Australia, including seven of the 20 predominant genotypes (WA-3, WA-10, WA-14, WA-24, WA-29 and WA-52) [14, 22, 39]. Six genotypes accounted for 71.6 % of the isolates (*n*=531/734). Fifty genotypes were unique to WA (64 %, *n*=50/78), including three dominant genotypes (WA-6, WA-51 and WA-63). Overall, approximately two-thirds of isolates were PLS with no co-resistances, with most isolates belonging to WA-10, WA-14, WA-24 and WA-29, which were confirmed by WGS to be equivalent to MLST-types ST11428, ST11864, ST7359 and ST7363, respectively.

WA-10 was frequently isolated on the east coast of Australia in 2012 and 2014 (known as iPLEX genotype G141 or NSW-6) [8, 39]. Although WA-10 accounted for approximately 5 % of cultured gonorrhoea cases in NSW in 2014, the genotype was infrequently identified in WA during the same time [8, 39]. However, within 3 years, WA-10 had become widely distributed in the Perth metropolitan region, i.e. urban WA, suggesting successful persistence in the community. WA-10 in our collection belongs to ST11428, which was first reported in the UK in 2011 [40], and also suggests international import for this genotype.

WA-14 has previously been reported as iPLEX genotype G88 or NSW-4, and in 2012 was the second most frequently identified genotype in Australia and was primarily associated with the MSM community in Qld, Victoria, SA, NSW and WA [8]. In 2017 in WA, WA-14 corresponding to ST11864 was mostly isolated from males (*n*=64/74) and was frequently associated with throat colonization (data not shown).

WA-24, previously reported as G122 or NSW-1 in 2012 and 2014 [39, 41], was the most frequently identified genotype in Australia in 2012 and was primarily associated with the heterosexual populations in Qld and WA [39, 41]. In a global context, phylogenetic analysis has revealed an association of ST7359 isolates, equivalent to WA-24, from NSW with isolates in Brighton, UK [40]. Comparison of the Western Australian ST7359 to the ST7359 WGS data available in PubMLST (over 4 000 *N *. *gonorrhoeae* records) showed fewer than 25 allelic differences in the core genome (*n*=1649 loci) of isolates from Brighton [9], Osaka, Japan (2015) [42], and Madrid, Spain (2016) (submitted to the database by Maria Dolores Guerrero, data not shown).

WA-29 of ST7363 was the predominant remote community genotype, and has previously been reported as G125. G125 was the most frequently identified genotype in Qld, SA, WA and Northern Territory in 2012 [22] and is associated with heterosexual Indigenous Australian populations residing in remote regions [6]. ST7363 in addition to ST11648, ST12040, ST12045 and ST12046 formed the BPG Aus1 while ST12042 comprised BPG Aus2, previously identified as unique genomic populations in remote community gonorrhoea infections [9]. In contrast to the urban/rural regions, which are characterized by diverse genotypes, gonorrhoea isolates from remote regions displayed a lack of diversity of genetic lineages over the 5 years between 2012 and 2017, suggesting BPGs Aus1 and Aus2 can stably persist over longer time frames in this population in the absence of importation of different genetic lineages. In addition, the lack of allelic diversity within BPGs Aus1 and Aus2 over the 5 years suggests the lineage is non-recombinogenic, a conclusion supported by BAPS comparison with 196 local isolates.

The integrated utilization of the Ng_cgc_400 scheme developed by Harrison *et al*. [32], in combination with the MLST, NG-MAST [30] and NG-STAR [31] typing schemes, improved the resolution of genetic lineages associated with AMR. A subset of the AMR isolates associated with Azi and Cef resistance were examined in detail. In 2017, azithromycin resistance was associated with WA-38 which formed BPG-3, WA 51/52 which formed BPG-4, and WA-10/12/13/14/15/16 which formed BPG-5. Three BPG-3 isolates of MLST ST1584 were collected from remote WA and were found to be closely related to Qld and SA PPNG/AziLR outbreak isolates [17]. Globally, the WA-38 isolates clustered in Ng_cgc_400_33 and this cluster contained a closely related PPNG/AziLR gonococcal isolate (PubMLST ID 31489) from Poland. This may indicate that the WA-38 PPNG/AziLR outbreak lineage could have been introduced to Australia sometime around 2010 via international travel. However, Australian gonococcal isolates in the PubMLST database are represented by 200 isolates from WA and 19 other isolates mostly from Qld, which limits genomic comparison at the national level. BPG-4 belonged to the Ng_cgc_400_325 cluster which contained isolates of European origin mostly from 2011 [40]. Lastly, BPG-5 isolates belonged to Ng_cgc_400_3 which represented a globally dispersed heterogeneous group circulating since 2014 [40, 42–45]. Locally, WA-13 was previously reported as Azi-G10 which caused an outbreak of 88 cases in NSW among the MSM population in 2017 [14]. WA-16 is similar to the AZI-G7 lineage in NSW which has an AziR phenotype. Although genotype WA-16 had at least one copy of the C2611T 23S RNA mutation by iPLEX typing, only one isolate expressed the AziR phenotype, which may suggest the local variant has fewer mutated 23S alleles than AZI-G7. In these instances, WGS revealed that the copy number of the mutated 23S alleles varied in a dose-dependent manner [46] and variably raised the MIC from 4 to 16 mg l^−1^ in strains from BPG-3, 4 and 5. Interestingly, AziR isolates harbouring A39T *mtrR* alone had MICs not exceeding 1 mg l^−1^, presumably due to the recent observation that this mutation does not affect MtrR binding to the *mtrCDE* promoter and is unlikely to result in induction of the efflux pump [47].

Since 2003, gonorrhoea in remote health regions has been treated with amoxicillin, probenecid and azithromycin, because oral treatments are logistically easier to administer in remote health services and due to the high rate of coincident chlamydia infection [48, 49]. Therapeutic guidelines have recently recommended a single dose of ceftriaxone. Cef DS isolates have been isolated in WA since 2008. Travel was a major contributor to Cef DS in WA, with four of the nine Cef DS isolates associated with interstate travel (WA-21, *n*=1) and international travel to Asia (WA-42, *n*=1) and the USA (WA-43, *n*=2). The first Cef DS isolate in a remote region was identified in the Kimberley in 2019 [50]. However, the mosaic *penA* allele commonly associated with Cef DS was not always present by iPLEX typing. The two WA21 isolates belonging to ST1901, originating in Japan [51], had the canonical mosaic *penA* allele while WA-41 of ST7827 had a *penA* allele containing the A501V mutation.

In conclusion, an in-depth analysis of the genomic epidemiology of gonococcal isolates in 2017 has shown that the increase in gonorrhoea cases was driven by AMS isolates associated with ST11428, ST11864, ST7359 and ST7363, and introduced via international travel with only a small number of observed cases in the urban/rural regions being associated with BPG Aus1 and Aus2 isolates. In addition, the appearance of AziLR isolates in remote communities was mostly associated with ingress of genetic lineages from urban/rural communities into remote regions, presumably associated with increased economic activity related to mining in the remote regions. These conclusions are supported by the 21 % reduction in gonorrhoea notification rates between 2019 and 2021 (Fig. 1) which occurred during the COVID-19 travel restrictions that prevented inter-state and international travel in and out of WA. With the re-opening of WA’s borders to inter-state and international travel in early 2022, enhanced surveillance strategies are crucial to ensure effectivity of first-line treatment in both remote and urban/rural regions of WA.

Mass parallel sequencing technologies have now been installed in surveillance laboratories and are now the gold standard for use in phylogenetic studies performed on cross-sectional studies such as this. However, iPLEX arrays are a very cost-effective means of parsing large libraries of isolates for AMR markers and should be considered a useful adjunct to daily surveillance operations. However, both technologies are best applied to pure cultures of *N. gonorrhoeae* which are difficult to obtain from remote areas of WA. Sampling from these jurisdictions are so low that models predict that it would take up to 3.5 years to detect AMR isolates by which stage these strains would be responsible for up to 20 % of cases per annum [52]. Further resourcing should be directed towards techniques for direct assessment of remote clinical samples via metagenomics sequencing or probe capture, as these samples do not provide live isolates for reliable genotyping and antibiogram testing.

## Supplementary Data

Supplementary material 1
