# Supplementary material for: Travel-associated lineages and unique endemic antimicrobial-susceptible lineages of Neisseria gonorrhoeae predominate in Western Australia
Source: Microb Genom. 2023 Mar 29;9(3):mgen000969. doi: 10.1099/mgen.0.000969 (PMC10132056; doi:10.1099/mgen.0.000969)
Supplement: Supplementary material 1 [file mgen-9-969-s001.pdf]

Travel associated lineages and unique endemic antimicrobial susceptible lineages of *Neisseria gonorrhoeae* predominate in Western Australia

Barakat A. Al Suwayyid[1,2], Ethan C. Haese[1], Shakeel Mowlaboccus[1,3,4], Julie C. Pearson[4], David Whiley[5], Paul Armstrong[6], Carolien Giele[6], Donna B. Mak[6,7], Lisa Bastian[6], Michael Wise[8], and Geoffrey W. Coombs[3,4], Charlene M. Kahler[1,9*].

[1]The Marshall Centre for Infectious Diseases Research and Training, The University of Western Australia, Crawley, Australia.

[2]Ministry of Education, Saudi Arabia.

[3]Antimicrobial Resistance and Infectious Diseases Research Laboratory, Murdoch University, Murdoch, Australia.

[4]Department of Microbiology, PathWest Laboratory Medicine-WA, Fiona Stanley Hospital, Murdoch, Australia.

[5] The University of Queensland Centre for Clinical Research (UQ-CCR), Faculty of Medicine, The University of Queensland, Brisbane, Queensland, Australia

[6]. Communicable Disease Control Directorate, Department of Health Western Australia

[7]. School of Medicine, University of Notre Dame Australia

[8]School of Physics, Mathematics and Computing, University of Western Australia

[9]. Telethon Kids Institute, Nedlands, Australia.

*Corresponding Author:

Email: charlene.kahler@uwa.edu.au

Supplementary Table 1: AGSP Interpretive Criteria for *N. gonorrhoeae* MIC values (Bell et al., 2013)

Antibiotic MIC (mg/L)	Sensitive	Less sensitive [a]	Resistant
**Ceftriaxone**	<0.06	0.6-0.25 (Cef DS)	Not defined [b]
**azithromycin**	<1.0	Not defined	>=1.0 (AziR) [c]
**Penicillin**	<0.06 (PHS)	0.6-0.5 (PLS)	>0.5
**Ciprofloxacin**	<0.06	0.6-0.5	>0.5 (CipR)
**Spectinomycin**	<=64	Not defined	>64.0
**Tetracycline**	<0.5	05-1.0	>1.0

[a] The term used to describe intermediate susceptibility in ceftriaxone is "decreased susceptibility".

[b] The absence or rare occurrence of an adequate number of evidence-based correlates between the MIC of isolates and treatment outcome means the breakpoint for resistance cannot yet be determined.

[c] The Centre for Disease Control (CDC), Atlanta, USA states that in the absence of established criteria, the use of critical MICs ≥1.0 mg/L to interpret the susceptibility of *N. gonorrhoeae* to this agent is recommended until more extensive assessments of clinical treatment outcome to this agent is available.

Supplementary Table 2: Summary of data for all WA-types of 734 *N. gonorrhoeae* strains isolated in WA as identified by *N. gonorrhoeae* iPLEX SNP typing.

Key:

Wt = wild-type locus not associated with AMR

X#Y = amino acid exchange of X for Y in position number (#) resulting in a known AMR phenotype

X- del = nucleotide (A/G/C/T) deletion associated with a known AMR phenotype

Y-insertion = nucleotide (A/G/C/T) insertion associated with a known AMR phenotype

Penicillin resistance = PonA1 (*ponA*1 is an allele of *ponA* that has a T to C transition at nucleotide 1261 of the ponA coding region), PBP2 (amino acid at position 501, 345A insertion, mosaic)

Azithromycin resistance = 23S rRNA nucleotide at positions 2059 and 2611, mtrR promoter mutations (A-del, T-insertion or meningococcal promoter), MtrR amino acid change (position 45)

Fluoroquinolones resistance = GyrA (amino acids at position 91 and 95)

iPLEX typing Scheme															WGS typing data	Other naming schemes (references)		
WA type (iPLEX-type)	Percentage of collection (n/734)	MLST by iPLEX	23S rRNA nucleotide 2059	23S rRNA nucleotide 2611	GyrA amino acid 91	GyrA amino acid 95	PonA1	*mtrR* promoter ?A deletion	-Meningo type*mtrR* ?	*mtrR* promoter T ?insertion	MtrR amino acid 45	PBP2 amino acid 501	PBP2 345A insertion	mosaic ?PBP2	MLST by WGS (n = number of sequenced isolates)	Genotype (C. Buckley et al., 2018)	NSW-type (M. M. Lahra et al., 2016)	Azi-type (David M Whiley, Kundu, et al., 2018)
WA-10	22.3%	11428	wt	wt	wt	wt	wt	wt	wt	wt	wt	wt	345A insertion	wt	ST11428 (n=8)	G141	NSW-6	
WA-14	10.0%	9363, 11420, 11463, 11864, 11900, 12396	wt	wt	wt	wt	wt	wt	wt	wt	wt	wt	345A insertion	wt	ST11864 (n=1)	G88	NSW-4	
WA-24	7.6%	7359, 7825, 7826	wt	wt	wt	wt	wt	wt	wt	wt	wt	wt	345A insertion	wt	ST7359 (n=1)	G122	NSW-1	
WA-29	7.6%	7363, 7832, 10243, 11052, 11656, 11659, 12083, 12546, 12906, 12959	wt	wt	wt	wt	L421P	wt	wt	wt	wt	wt	345A insertion	wt	ST7363 (n=47)	G125	NSW-32	
WA-03	5.5%	1587, 11723	wt	wt	S91F	D95A	wt	wt	wt	wt	wt	wt	345A insertion	wt	ST1587 (n=1)		NSW-118	
WA-06	4.8%	8135, 11997	wt	wt	wt	wt	wt	wt	wt	wt	wt	wt	wt	wt	ST8135 (n=1)			
WA-32	4.0%	1583, 8156, 9361, 12078, 12246, 12503, 12529	wt	wt	wt	wt	L421P	A-del	wt	wt	wt	wt	345A insertion	wt	ST8156 (n=1)		NSW-34	
WA-52	3.7%	1179, 1596, 11673, 11993	wt	C2611T	wt	wt	wt	wt	wt	wt	wt	wt	wt	wt	ST1596 (n=2)			AZI-G2
WA-63	3.4%	12046	wt	wt	wt	wt	L421P	wt	wt	wt	wt	wt	345A insertion	wt	ST12046 (n=20)			
WA-51	3.4%	1179, 1596, 11673, 11993	wt	wt / C2611T	wt	wt	wt	wt	wt	wt	wt	wt	wt	wt	ST1596 (n=4)			
WA-22	2.5%	1579, 1896, 11194, 12091, 12907	wt	wt	wt	wt	L421P	A-del	wt	wt	wt	wt	345A insertion	wt	ST12042 (n=1, EXNG662)	G115	NSW-8	
WA-30	2.4%	7363, 7832, 10243, 11052, 11656, 11659, 12083, 12546, 12906, 12959	wt	wt	S91F	D95A	wt	wt	wt	wt	wt	wt	345A insertion	wt	ST7363 (n=1)			
WA-56	2.0%	11413, 12042	wt	wt	wt	wt	L421P	wt	wt	wt	wt	wt	345A insertion	wt	12042 (n=11)			
WA-27	1.6%	8143	wt	wt	S91F	D95A	wt	wt	wt	wt	wt	wt	345A insertion	wt	ST8143 (n=1)		NSW-19	
WA-47	1.0%	8122, 8138, 12007	wt	wt	wt	wt	wt	wt	wt	T-insertion	wt	wt	345A insertion	wt	ST8122 (n=1)		NSW-66	
WA-16	0.9%	9363, 11420, 11463, 11864, 11900, 12396	wt	wt / C2611T	wt	wt	wt	wt	wt	wt	wt	wt	345A insertion	wt	ST9363 (n=2)			AZI-G7

WA type (iPLEX-type)	Percentage of collection (n/734)	Predicted MLST	23S rRNA nucleotide 2059	23S rRNA nucleotide 2611	GyrA amino acid 91	GyrA amino acid 95	PonA1	*mtrR* promoter ?A deletion	-Meningo type*mtrR* ?	*mtrR* promoter T ?insertion	MtrR amino acid 45	PBP2 amino acid 501	PBP2 345A insertion	mosaic ?PBP2	MLST by WGS (n = number of sequenced isolates)	Genotype (C. Buckley et al., 2018)	NSW-type (M. M. Lahra et al., 2016)	Azi-type (David M Whiley, Kundu, et al., 2018)
WA-59	0.8%	12040	wt	wt	wt	wt	L421P	wt	wt	wt	wt	wt	345A insertion	wt	ST12040 (n=4)			
WA-62	0.8%	7365, 11707, 11709, 12045, 12978	wt	wt	wt	wt	L421P	wt	wt	T-insertion	wt	wt	345A insertion	wt	ST12045 (n=1)			
WA-49	0.7%	1179, 1596, 11673, 11993	wt	wt	wt	wt	wt	wt	wt	wt	wt	wt	wt	wt	ST1596 (n=1)	G91	NSW-18	
WA-38	0.7%	1584, 8128, 8145, 10242, 11418, 11698, 11719, 12492, 12505, 12547	wt	C2611T	wt	wt	wt	wt	wt	wt	wt	wt	345A insertion	wt	ST1584 (n=5)		NSW-44	AZI-1
WA-58	0.5%	10316, 11685, 11699, 11700, 12527	wt	wt	S91F	D95A	wt	wt	wt	wt	wt	wt	345A insertion	wt	ST7363 (n=1/ EXNG863)			
WA-13	0.5%	9363, 11420, 11463, 11864, 11900, 12396	wt	wt	wt	wt	wt	wt	Meningo mtrF	wt	wt	wt	345A insertion	wt	ST9363 (n=3)			AZI-G10
WA-19	0.5%	novel2	wt	wt	S91F	D95A	wt	wt	wt	wt	wt	wt	345A insertion	wt	ST13480 (n=1)			
WA-20	0.5%	1901, 6814, 9365, 9751, 10312, 10315, 10884, 10885, 10886, 10887, 10888, 10889, 10890, 10891, 10892, 10894, 10896, 10897, 10898, 11107, 11232, 11427, 11602, 11981, 11992, 12088, 12517, 12519, 12530, 12534, 12538, 12539, 12540, 12541, 12542, 12544	wt	wt	S91F	D95G	L421P	A-del	wt	wt	wt	wt	345A insertion	wt	ST11981 (n=1)		NSW-23	

WA type (iPLEX-type)	Percentage of collection (n/734)	Predicted MLST	23S rRNA nucleotide 2059	23S rRNA nucleotide 2611	GyrA amino acid 91	GyrA amino acid 95	PonA1	*mtrR* promoter ?A deletion	-Meningo type*mtrR* ?	*mtrR* promoter T ?insertion	MtrR amino acid 45	PBP2 amino acid 501	PBP2 345A insertion	mosaic ?PBP2	MLST by WGS (n = number of sequenced isolates)	Genotype (C. Buckley et al., 2018)	NSW-type (M. M. Lahra et al., 2016)	Azi-type (David M Whiley, Kundu, et al., 2018)
WA-21	0.4%	1901, 6814, 9365, 9751, 10312, 10315, 10884, 10885, 10886, 10887, 10888, 10889, 10890, 10891, 10892, 10894, 10896, 10897, 10898, 11107, 11232, 11427, 11602, 11981, 11992, 12088, 12517, 12519, 12530, 12534, 12538, 12539, 12540, 12541, 12542, 12544	wt	wt	S91F	D95G	L421P	A-del	wt	wt	wt	wt	wt	mosiac	one isolate sequenced ST1901	G117	NSW-3	
WA-40	0.4%	7827, 9324, 11182, 11976, 11995, 12545	wt	wt	wt	wt	L421P	A-del	wt	wt	G45D	wt	345A insertion	wt	ST7827 (n=1)			
WA-41	0.4%	7827, 9324, 11182, 11976, 11995, 12545	wt	wt	S91F	D95A	L421P	A-del	wt	wt	G45D	A501V	345A insertion	wt	ST7827 (n=1)			
WA-42	0.4%	7827, 9324, 11182, 11976, 11995, 12545	wt	wt	S91F	D95G	L421P	A-del	wt	wt	G45D	A501V	345A insertion	wt	ST7827 (n=1)			
WA-43	0.4%	6962, 11648	wt	wt	wt	wt	L421P	wt	wt	wt	wt	wt	345A insertion	wt	ST11648 (n=3)			
WA-55	0.2%	1588, 11966	wt	wt	S91F	D95A	L421P	wt	wt	wt	wt	wt	345A insertion	wt	ST15607 (n=1)	G5	NSW-2	
WA-61	0.2%	7365, 11707, 11709, 12045, 12978	wt	wt	wt	wt	L421P	wt	wt	wt	wt	wt	345A insertion	wt	ST12045 (n=1)	G36		
WA-01	0.2%	11184	wt	wt	S91F	D95G	wt	wt	wt	wt	wt	wt	345A insertion	wt	ST11184 (n=1)			
WA-07	0.2%	8776	wt	wt	S91F	D95G	wt	wt	wt	wt	wt	wt	345A insertion	wt	ST8776 (n=1)			
WA-12	0.2%	11428	wt	C2611T	wt	wt	wt	wt	wt	wt	wt	wt	345A insertion	wt	ST11428 (n=2)			
WA-31	0.2%	7363, 7832, 10243, 11052, 11656, 11659, 12083, 12546, 12906, 12959	wt	wt	S91F	D95G	L421P	A-del	wt	wt	wt	wt	wt	mosiac	ST7363 (n=1)			
WA-34	0.2%	1583, 8156, 9361, 12078, 12246, 12503, 12529	wt	wt	S91F	D95G	wt	wt	wt	wt	wt	wt	345A insertion	wt	ST1583 (n=1)		NSW-96	

WA type (iPLEX-type)	Percentage of collection (n/734)	Predicted MLST	23S rRNA nucleotide 2059	23S rRNA nucleotide 2611	GyrA amino acid 91	GyrA amino acid 95	PonA1	*mtrR* promoter ?A deletion	-Meningo type*mtrR* ?	*mtrR* promoter T ?insertion	MtrR amino acid 45	PBP2 amino acid 501	PBP2 345A insertion	mosaic ?PBP2	MLST by WGS (n = number of sequenced isolates)	Genotype (C. Buckley et al., 2018)	NSW-type (M. M. Lahra et al., 2016)	Azi-type (David M Whiley, Kundu, et al., 2018)
WA-54	0.2%	8133	wt	wt	S91F	D95A	L421P	wt	wt	wt	wt	wt	345A insertion	wt	ST8133 (n=1)			
WA-60	0.2%	1600, 12000	wt	wt	wt	wt	wt	wt	wt	wt	wt	wt	345A insertion	wt	ST1600 (n=1)			
WA-67	0.2%	7363, 7832, 10243, 11052, 11656, 11659, 12083, 12546, 12906, 12959	wt	wt	S91F	D95A	wt	wt	wt	wt	wt	wt	wt	mosiac	ST7363 (n=1)			
WA-71	0.2%	1599, 1931, 10934, 11183, 11191, 11994	wt	wt	S91F	wt	L421P	wt	wt	wt	G45D	A501V	345A insertion	wt	ST11191 (n=1)			
WA-75	0.2%	1179, 1596, 11673, 11993	wt	wt	wt	wt	L421P	wt	wt	wt	wt	wt	345A insertion	wt	ST1596 (n=2)			
WA-77	0.2%	1600, 12000	wt	wt	S91F	D95A	L421P	A-del	wt	wt	wt	A501V	345A insertion	wt	ST1600 (n=1)		NSW-123	
WA-02	0.1%	8775	wt	wt	S91F	D95A	wt	wt	wt	wt	wt	wt	345A insertion	wt	ST8775 (n=1)			
WA-04	0.1%	10622	wt	wt	S91F	D95A	wt	wt	wt	wt	wt	wt	345A insertion	wt	ST10622 (n=1)			
WA-05	0.1%	11965	wt	wt	S91F	D95G	wt	wt	wt	wt	wt	wt	345A insertion	wt	ST11965 (n=1)			
WA-08	0.1%	1580, 1923, 11999, 12533	wt	wt	wt	wt	wt	wt	wt	wt	wt	wt	345A insertion	wt	ST11999 (n=1)			
WA-09	0.1%	1580, 1923, 11999, 12533	wt	C2611T	wt	wt	wt	wt	wt	wt	G45D	wt	345A insertion	wt	ST1580 (n=1)			AZI-G6
WA-11	0.1%	11428	wt	wt / C2611T	wt	wt	wt	wt	wt	wt	wt	wt	345A insertion	wt	ST11428 (n=1)			
WA-15	0.1%	9363, 11420, 11463, 11864, 11900, 12396	wt	wt	S91F	D95A	wt	wt	Meningo mtrR	wt	wt	wt	345A insertion	wt	ST9363 (n=1)			AZI-G9
WA-17	0.1%	9362	wt	wt	S91F	D95A	wt	A-del	wt	wt	wt	wt	345A insertion	wt	ST9362 (n=1)			
WA-18	0.1%	novel1	wt	wt	S91F	D95G	wt	wt	wt	wt	wt	wt	345A insertion	wt	ST14418 (n=1)			
WA-23	0.1%	1902, 8131, 10313, 12501	wt	wt	S91F	D95A	L421P	wt	wt	wt	wt	A501T	345A insertion	wt	no isolates sequenced			
WA-25	0.1%	7359, 7825, 7826	wt	wt	wt	wt	wt	A-del	wt	wt	wt	wt	345A insertion	wt	ST12046 (n=1, EXNG855)			
WA-26	0.1%	9901, 11998	wt	wt	S91F	D95G	wt	wt	wt	wt	wt	wt	345A insertion	wt	ST9901 (n=1)			
WA-28	0.1%	8123	wt	wt	S91F	D95A	L421P	A-del	wt	wt	wt	A501T	345A insertion	wt	ST8123 (n=1)			
WA-33	0.1%	1583, 8156, 9361, 12078, 12246, 12503, 12529	wt	wt	S91F	D95A	L421P	A-del	wt	wt	wt	wt	345A insertion	wt	ST1583 (n=1)			

WA type (iPLEX-type)	Percentage of collection (n/734)	Predicted MLST	23S rRNA nucleotide 2059	23S rRNA nucleotide 2611	GyrA amino acid 91	GyrA amino acid 95	PonA1	*mtrR* promoter ?A deletion	-Meningo type*mtrR* ?	*mtrR* promoter T ?insertion	MtrR amino acid 45	PBP2 amino acid 501	PBP2 345A insertion	mosaic ?PBP2	MLST by WGS (n = number of sequenced isolates)	Genotype (C. Buckley et al., 2018)	NSW-type (M. M. Lahra et al., 2016)	Azi-type (David M Whiley, Kundu, et al., 2018)
WA-35	0.1%	1583, 8156, 9361, 12078, 12246, 12503, 12529	wt	wt	S91F	D95G	wt	A-del	wt	wt	wt	wt	345A insertion	wt	ST1583 (n=1)		NSW-31	
WA-36	0.1%	1583, 8156, 9361, 12078, 12246, 12503, 12529	wt	wt / C2611T	wt	wt	L421P	A-del	wt	wt	wt	wt	345A insertion	wt	ST8146 (n=1)			AZI-G3
WA-37	0.1%	11172, 11690	wt	wt	wt	wt	wt	wt	wt	wt	wt	wt	345A insertion	wt	ST11172 (n=1)			
WA-39	0.1%	1599, 1931, 10934, 11183, 11191, 11994	wt	wt	wt	wt	wt	wt	wt	wt	wt	wt	345A insertion	wt	ST1599 (n=1)			
WA-44	0.1%	novel3	wt	wt	wt	wt	wt	wt	wt	wt	wt	wt	345A insertion	wt	ST15331 (n=1)			
WA-45	0.1%	8112	wt	wt	S91F	D95A	wt	wt	wt	wt	wt	wt	345A insertion	wt	ST8122 (n=1)			
WA-46	0.1%	1595, 1906, 5688, 7824, 11255, 11260, 11695, 11717, 12081	wt	wt	wt	wt	wt	wt	wt	wt	wt	wt	345A insertion	wt	ST11260 (n=1)			
WA-48	0.1%	novel4	wt	wt	S91F	D95A	wt	wt	wt	wt	wt	wt	345A insertion	wt	ST13266 (n=1)			
WA-50	0.1%	1179, 1596, 11673, 11993	wt	wt	S91F	D95A	L421P	wt	wt	wt	wt	wt	345A insertion	wt	ST1596 (n=1)			
WA-53	0.1%	8133	wt	wt	wt	wt	L421P	wt	wt	wt	wt	wt	345A insertion	wt	ST8133 (n=1)			
WA-57	0.1%	10316, 11685, 11699, 11700, 12527	wt	wt	wt	wt	wt	wt	wt	wt	wt	wt	345A insertion	wt	ST10316 (n=1)			
WA-64	0.1%	1903, 11711, 11712	wt	wt	wt	wt	wt	wt	wt	wt	wt	wt	345A insertion	wt	ST15608 (n=1)			
WA-65	0.1%	1601	wt	wt	wt	wt	wt	wt	wt	wt	G45D	wt	345A insertion	wt	ST1601 (n=1)		NSW-124	
WA-66	0.1%	1580, 1923, 11999, 12533	wt	wt	wt	wt	wt	wt	wt	wt	G45D	wt	345A insertion	wt	ST1580 (n=1)			
WA-68	0.1%	7363, 7832, 10243, 11052, 11656, 11659, 12083, 12546, 12906, 12959	wt	wt	wt	wt	wt	wt	wt	T-insertion	wt	wt	345A insertion	wt	ST1890 (n=1, EXNG873)		NSW-47	
WA-69	0.1%	9903, 11683	wt	wt	S91F	D95A	wt	wt	wt	wt	wt	wt	345A insertion	wt	ST9903 (n=1)			
WA-70	0.1%	1583, 8156, 9361, 12078, 12246, 12503, 12529	wt	wt	S91F	D95A	wt	wt	wt	wt	wt	A501V	345A insertion	wt	ST1583 (n=1)			

WA type (iPLEX-type)	Percentage of collection (n/734)	Predicted MLST	23S rRNA nucleotide 2059	23S rRNA nucleotide 2611	GyrA amino acid 91	GyrA amino acid 95	PonA1	*mtrR* promoter ?A deletion	-Meningo type*mtrR* ?	*mtrR* promoter T ?insertion	MtrR amino acid 45	PBP2 amino acid 501	PBP2 345A insertion	mosaic ?PBP2	MLST by WGS (n = number of sequenced isolates)	Genotype (C. Buckley et al., 2018)	NSW-type (M. M. Lahra et al., 2016)	Azi-type (David M Whiley, Kundu, et al., 2018)
WA-72	0.1%	6962, 11648	wt	wt	S91F	D95A	wt	wt	wt	wt	wt	wt	345A insertion	wt	ST11648 (n=1)			
WA-73	0.1%	novel4	wt	wt	wt	wt	wt	wt	wt	wt	wt	wt	345A insertion	wt	ST123913 (n=1)			
WA-74	0.1%	6808	wt	wt	S91F	D95G	wt	wt	wt	wt	wt	wt	345A insertion	wt	ST6808 (n=1)			
WA-76	0.1%	8780	wt	wt	S91F	D95A	L421P	wt	wt	wt	wt	wt	345A insertion	wt	ST8780 (n=1)		NSW-137	
WA-78	0.1%	1600, 12000	wt	wt	wt	wt	wt	wt	wt	wt	wt	wt	345A insertion	wt	ST1600 (n=1)		NSW-36	

Supplementary Table 3: Summary of whole genome sequencing data of 254 Australian gonococcal isolates used in the study.

Key:

- = partial sequence in the contigs such that a sequence type was not defined

NP = not present (no matching sequence in the contigs)

NA = not assessed

NG = Not grouped by BAPs

ND = sequence type was not defined (novel ST) even though all alleles were identified separately.

M-series = isolates from Queensland (donated by Professor David Whiley)

ExNG series = isolates collected in Western Australia (as described in methods).

Antibiogram outcomes for penicillin (PEN), ceftriaxone (CEF), ciprofloxacin (CIP), tetracycline (TET), spectinomycin (SPEC) and azithromycin (AZI) were determined as per the instructions in Supplementary Table 1.

Isolate	PubMLST ID	PEN	CEF	CIP	TET	SPEC	AZI	NG-MAST	NG-STAR	MLST	WA type	BAP group	Ng_cgc_400	pST (conjugative)	pST (beta lactamase)	pST (cryptic)	Reference
EXNG202	45011	LS	S	S	Not TRNG	S	S	1498	829	12045	NA	BPG-1	379	NP	NP	-	[9]
EXNG204	45012	LS	S	S	Not TRNG	S	S	10103	843	7363	NA	BPG-1	380	NP	NP	-	[9]
EXNG206	45015	-	-	-	-	-	-	10080	842	7363	NA	BPG-1	380	NP	NP	-	[9]
EXNG209	45016	LS	S	S	TRNG	S	S	9716	827	12042	NA	BPG-2	381	-	NP	33	[9]
EXNG210	45017	LS	S	S	Not TRNG	S	S	7126	839	12040	NA	BPG-1	380	NP	NP	-	[9]
EXNG213	45018	LS	S	S	Not TRNG	S	S	7126	839	12040	NA	BPG-1	380	NP	NP	-	[9]
EXNG214	45020	LS	S	S	Not TRNG	S	S	758	1552	12045	NA	BPG-1	379	NP	NP	41	[9]
EXNG217	45046	LS	S	S	Not TRNG	S	S	758	755	12045	NA	BPG-1	379	NP	NP	41	[9]
EXNG218	45047	LS	S	S	Not TRNG	S	S	10103	843	7363	NA	BPG-1	380	NP	NP	-	[9]
EXNG219	45019	LS	S	S	Not TRNG	S	S	10103	843	7363	NA	BPG-1	380	NP	NP	-	[9]
EXNG224	45048	LS	S	S	Not TRNG	S	S	7803	754	12040	NA	BPG-1	380	NP	NP	-	[9]
EXNG225	45021	LS	S	S	Not TRNG	S	S	758	755	12045	NA	BPG-1	379	NP	NP	-	[9]
EXNG226	45022	PPNG	S	R	TRNG	S	S	436	830	8145	NA	NG	184	-	-	9	[9]
EXNG229	45029	LS	S	S	Not TRNG	S	S	758	755	12045	NA	BPG-1	379	NP	NP	-	[9]
EXNG230	45030	LS	S	S	TRNG	S	S	9716	827	12042	NA	BPG-2	381	-	NP	-	[9]
EXNG232	45031	CMRP	S	HLR	Not TRNG	S	S	225	177	1901	NA	NG	18	NP	NP	-	[9]
EXNG234	45049	LS	S	S	Not TRNG	S	S	758	755	12045	NA	BPG-1	379	NP	NP	-	[9]
EXNG235	45050	PPNG	S	S	TRNG	S	S	10105	837	1924	NA	NG	179	-	-	-	[9]
EXNG236	45051	LS	S	S	Not TRNG	S	S	758	755	12045	NA	BPG-1	379	NP	NP	-	[9]
EXNG237	45037	CMRP	DS	HLR	Not TRNG	S	S	1407	90	7360	NA	NG	3	NP	NP	2	[9]
EXNG238	45038	PPNG	S	HLR	TRNG	S	S	10114	831	1588	NA	NG	21	-	-	32	[9]
EXNG239	45027	LS	S	S	Not TRNG	S	S	7206	840	7363	NA	BPG-1	380	NP	-	-	[9]
EXNG242	45035	LS	S	S	Not TRNG	S	S	4186	231	7359	NA	NG	177	NP	NP	-	[9]
EXNG248	45052	LS	S	S	Not TRNG	S	S	4186	231	7359	NA	NG	177	NP	NP	-	[9]
EXNG250	45053	LS	S	S	Not TRNG	S	S	4768	178	1584	NA	BPG-3	33	-	NP	ND	[9]
EXNG252	45036	LS	S	S	Not TRNG	S	S	758	755	12045	NA	BPG-1	379	NP	NP	-	[9]
EXNG253	45054	LS	S	S	Not TRNG	S	S	7126	839	12040	NA	BPG-1	380	NP	NP	-	[9]
EXNG254	45055	LS	S	S	Not TRNG	S	S	10117	754	7363	NA	BPG-1	380	NP	NP	-	[9]
EXNG257	45039	PPNG	S	HLR	TRNG	S	S	10106	73	7822	NA	NG	29	-	-	55	[9]
EXNG259	45056	LS	S	S	Not TRNG	S	S	7126	839	12040	NA	BPG-1	380	NP	NP	-	[9]
EXNG261	45057	LS	S	S	TRNG	S	S	9716	827	12042	NA	BPG-2	381	ND	NP	-	[9]
EXNG264	45058	LS	S	S	TRNG	S	S	9716	827	12042	NA	BPG-2	381	-	NP	-	[9]
EXNG266	45059	LS	S	S	Not TRNG	S	S	7206	840	7363	NA	BPG-1	380	NP	NP	33	[9]
EXNG270	45040	LS	S	HLR	Not TRNG	S	S	5267	73	7822	NA	NG	29	-	NP	-	[9]
EXNG271	45060	LS	S	S	TRNG	S	S	9716	827	12042	NA	BPG-2	381	-	NP	-	[9]
EXNG272	45061	LS	S	S	Not TRNG	S	S	10117	754	7363	NA	BPG-1	380	NP	NP	-	[9]
EXNG277	45062	LS	S	S	Not TRNG	S	S	19435	840	12044	NA	BPG-1	383	NP	NP	-	[9]
EXNG278	45063	LS	S	S	Not TRNG	S	S	8063	829	12046	NA	BPG-1	379	-	NP	ND	[9]
EXNG281	45041	CMRP	DS	HLR	Not TRNG	S	S	4822	90	1901	NA	NG	3	NP	NP	-	[9]
EXNG282	45064	LS	S	S	Not TRNG	S	S	7126	829	12045	NA	BPG-1	379	NP	NP	-	[9]
EXNG283	45042	PPNG	S	HLR	Not TRNG	S	S	8842	836	1890	NA	NG	21	NP	-	-	[9]
EXNG285	45065	LS	S	S	Not TRNG	S	S	758	755	12045	NA	BPG-1	379	NP	NP	-	[9]
EXNG287	45043	CMRP	S	HLR	TRNG	S	S	10109	715	11249	NA	NG	61	273	-	-	[9]
EXNG288	45045	LS	S	R	TRNG	S	S	9716	827	12042	NA	BPG-2	381	-	NP	-	[9]
EXNG289	45066	LS	S	S	Not TRNG	S	S	7206	841	7363	NA	BPG-1	380	274	NP	-	[9]
EXNG290	45067	LS	S	S	Not TRNG	S	S	8022	844	12046	NA	BPG-1	379	274	NP	-	[9]
EXNG294	45068	LS	S	S	TRNG	S	S	9716	827	12042	NA	BPG-2	381	-	NP	ND	[9]
EXNG295	45069	PPNG	S	S	Not TRNG	S	S	5268	178	10317	NA	BPG-3	33	-	-	-	[9]
EXNG296	45070	LS	S	S	TRNG	S	S	9716	827	12042	NA	BPG-2	381	-	NP	-	[9]
EXNG301	45032	LS	S	S	Not TRNG	S	S	10125	838	12041	NA	NG	382	-	NP	ND	[9]
EXNG302	45071	LS	S	S	Not TRNG	S	S	7268	832	8156	NA	NG	17	NP	NP	-	[9]
EXNG304	45044	LS	S	HLR	Not TRNG	S	S	5533	90	1901	NA	NG	3	NP	NP	-	[9]
EXNG305	45033	LS	S	S	Not TRNG	S	S	8022	844	12046	NA	BPG-1	379	NP	NP	ND	[9]
EXNG307	45072	PPNG	S	S	Not TRNG	S	S	5268	178	10317	NA	BPG-3	33	-	-	-	[9]
EXNG309	45073	CMRP	S	S	Not TRNG	S	S	21	139	1579	NA	NG	24	NP	NP	-	[9]
EXNG314	45074	LS	S	S	Not TRNG	S	S	9716	827	12043	NA	BPG-2	381	-	NP	-	[9]
EXNG316	45075	LS	S	S	Not TRNG	S	S	4186	231	7359	NA	NG	177	NP	NP	-	[9]
EXNG321	45034	PPNG	S	HLR	TRNG	S	HLR	14850	202	12039	NA	NG	29	272	-	-	[9]
EXNG322	45330	LS	S	S	Not TRNG	S	S	10120	85	8149	NA	NG	384	-	NP	ND	[9]
EXNG323	54498	S	-	-	-	-	R	17048	1994	1596	NA	BPG-4	325	231	NP	15	[9]
EXNG324	46029	S	S	S	Not HLR	S	R	17047	1994	1596	WA-52	BPG-4	325	231	NP	-	This study
EXNG325	46035	S	-	-	-	-	S	5031	307	1596	NA	BPG-4	325	275	NP	-	This study
EXNG326	46036	S	-	-	-	-	S	5031	307	1596	NA	BPG-4	325	275	NP	15	This study
EXNG327	46037	S	-	-	-	-	S	19436	307	1596	NA	BPG-4	325	275	NP	-	This study
EXNG328	46039	S	-	-	-	-	S	387	729	8135	NA	NG	304	54	NP	27	This study
EXNG329	46044	S	-	-	-	-	R	17047	1994	1596	NA	BPG-4	325	231	NP	-	This study
EXNG330	46046	S	S	S	Not HLR	S	R	19437	1028	1596	WA-52	BPG-4	325	231	NP	-	This study
EXNG331	57124	S	-	-	-	-	R	17047	1028	1596	NA	BPG-4	325	-	NP	-	This study

Isolate	PubMLST ID	PEN	CEF	CIP	TET	SPEC	AZI	NG-MAST	NG-STAR	MLST	WA type	BAP group	Ng_cgc_ 400	pST (conjugative)	pST (beta lactamase)	pST (cryptic)	Reference
EXNG332	57125	S	S	S	Not HLR	S	S	387	729	8135	WA-06	NG	304	-	NP	-	This study
EXNG335	93429	LS	S	S	S	S	S	ND	NA	11428	WA-10	BPG-5	3	NP	NP	3	This study
EXNG338	63043	LS	S	S	Not HLR	S	S	8022	844	12046	WA-63	BPG-1	379	NP	NP	NP	This study
EXNG345	63044	LS	S	S	Not HLR	S	S	18030	1245	7363	WA-29	BPG-1	NA	NP	NP	33	This study
EXNG347	63045	LS	S	S	Not HLR	S	S	8022	844	12046	WA-63	BPG-1	379	NP	NP	NP	This study
EXNG349	77410	LS	S	S	Not HLR	S	S	6339	NA	1601	WA-65	NA	24	NP	NP	4	This study
EXNG351	63046	LS	S	S	Not HLR	S	S	9716	1968	11648	WA-43	BPG-1	NA	NP	NP	-	This study
EXNG352	77411	LS	S	S	Not HLR	S	S	292	NA	8122	WA-47	NA	321	-	NP	25	This study
EXNG353	94160	LS	S	S	Not HLR	S	S	5802	1034	9363	WA-13	BPG-5	3	NP	NP	-	This study
EXNG359	63047	LS	S	S	HLR	S	S	9716	827	12042	WA-56	BPG-2	381	-	NP	-	This study
EXNG364	94161	LS	S	S	Not HLR	S	R	-	1368	1580	WA-09	BPG-5	3	NP	NP	-	This study
EXNG366	77412	PPNG	S	R	Not HLR	S	S	5624	NA	8143	WA-27	NA	25	-	-	26	This study
EXNG370	63048	LS	S	S	Not HLR	S	S	-	1245	7363	WA-29	BPG-1	NA	NP	NP	-	This study
EXNG371	63049	LS	S	S	Not HLR	S	S	ND	754	12040	WA-59	BPG-1	380	NP	NP	-	This study
EXNG375	77413	PPNG	S	HLR	HLR	S	S	-	NA	8133	WA-54	NA	21	385	1	32	This study
EXNG376	63050	LS	S	S	Not HLR	S	S	8022	844	12046	WA-63	BPG-1	379	NP	NP	NP	This study
EXNG378	63051	LS	S	S	Not HLR	S	S	ND	840	7363	WA-29	BPG-1	380	NP	NP	-	This study
EXNG387	77414	PPNG	S	R	HLR	S	S	-	NA	15607	WA-55	NA	21	386	-	32	This study
EXNG390	63052	LS	S	S	Not HLR	S	S	8022	844	13917	WA-63	BPG-1	379	NP	NP	-	This study
EXNG391	94162	LS	S	LS	Not HLR	S	S	-	NA	11864	WA-14	BPG-5	3	NP	NP	-	This study
EXNG395	63053	LS	S	S	Not HLR	S	S	8022	844	12046	WA-63	BPG-1	379	NP	NP	NP	This study
EXNG402	63054	LS	S	S	Not HLR	S	S	ND	1245	7363	WA-29	BPG-1	NA	NP	NP	-	This study
EXNG403	93428	S	S	S	Not HLR	S	R	17047	1028	1596	WA-51	BPG-4	325	-	NP	-	This study
EXNG414	77415	LS	S	S	Not HLR	S	S	14032	NA	7827	WA-40	NA	175	NP	NP	23	This study
EXNG420	77416	LS	S	S	HLR	S	S	11461	NA	1599	WA-39	NA	111	-	NP	-	This study
EXNG421	63055	LS	S	S	Not HLR	S	S	18030	1245	7363	WA-29	BPG-1	380	NP	NP	-	This study
EXNG422	77417	CMRP	DS	HLR	Not HLR	S	S	-	NA	7827	WA-41	NA	175	NP	NP	-	This study
EXNG425	77418	LS	S	HLR	HLR	S	S	ND	NA	11184	WA-01	NA	NA	-	NP	-	This study
EXNG430	63056	LS	S	S	Not HLR	S	S	-	754	7363	WA-29	BPG-1	NA	NP	NP	33	This study
EXNG438	63057	LS	S	S	Not HLR	S	S	18030	1245	7363	WA-29	BPG-1	380	NP	NP	-	This study
EXNG440	93427	LS	S	LS	Not HLR	S	R	5802	1034	9363	WA-13	BPG-5	3	NP	NP	-	This study
EXNG453	63058	LS	S	S	Not HLR	S	S	8022	844	12046	WA-63	BPG-1	379	NP	NP	-	This study
EXNG456	63059	LS	S	S	Not HLR	S	S	8022	844	12046	WA-63	BPG-1	NA	NP	NP	NP	This study
EXNG457	63060	LS	S	S	HLR	S	S	9716	827	12042	WA-56	BPG-2	NA	-	NP	-	This study
EXNG458	63061	LS	S	S	HLR	S	S	9716	827	12042	WA-56	BPG-2	381	-	NP	-	This study
EXNG463	63062	LS	S	S	Not HLR	S	S	-	754	7363	WA-29	BPG-1	NA	NP	NP	-	This study
EXNG464	63063	LS	S	S	Not HLR	S	S	11514	NA	11648	WA-43	BPG-1	NA	NP	NP	-	This study
EXNG467	63064	LS	S	S	HLR	S	S	ND	NA	11255	WA-46	NG	NA	-	NP	-	This study
EXNG468	63065	LS	S	S	Not HLR	S	S	ND	754	7363	WA-29	BPG-1	380	NP	NP	-	This study
EXNG470	77419	PPNG	S	HLR	HLR	S	S	ND	NA	14418	WA-18	NA	25	-	-	-	This study
EXNG475	63066	LS	S	S	HLR	S	S	-	1245	7363	NA	NA	NA	NP	NP	-	This study
EXNG476	88781	LS	S	S	Not HLR	S	S	-	63	11428	WA-10	BPG-5	3	NP	NP	-	This study
EXNG478	94163	LS	S	S	Not HLR	S	R	-	62	11428	WA-12	BPG-5	3	NP	NP	3	This study
EXNG480	63067	LS	S	S	Not HLR	S	S	-	1245	7363	WA-29	BPG-1	380	NP	NP	-	This study
EXNG482	77420	LS	S	HLR	HLR	S	S	ND	NA	8112	WA-45	NA	NA	387	NP	-	This study
EXNG484	63068	LS	S	S	Not HLR	S	S	9920	754	7363	WA-29	BPG-1	NA	NP	NP	-	This study
EXNG493	63069	LS	S	S	Not HLR	S	S	-	NA	7363	WA-29	BPG-1	NA	NP	NP	ND	This study
EXNG495	63070	LS	S	S	Not HLR	S	S	ND	754	7363	WA-29	BPG-1	380	NP	NP	-	This study
EXNG501	94164	LS	S	S	Not HLR	S	S	8709	1997	9363	WA-16	BPG-5	3	NP	NP	-	This study
EXNG505	63071	LS	S	S	Not HLR	S	S	-	1245	7363	WA-29	BPG-1	NA	NP	NP	-	This study
EXNG510	63072	LS	S	S	Not HLR	S	S	8022	844	12046	WA-63	BPG-1	379	NP	NP	NP	This study
EXNG512	63073	LS	S	S	Not HLR	S	S	-	NA	7363	WA-29	BPG-1	380	NP	NP	-	This study
EXNG516	63074	LS	S	S	Not HLR	S	S	-	755	12045	WA-61	BPG-1	379	NP	NP	-	This study
EXNG517	94165	LS	S	S	Not HLR	S	S	8709	63	9363	WA-16	BPG-5	3	NP	NP	-	This study
EXNG522	77421	LS	S	S	Not HLR	S	S	4990	NA	1600	WA-60	NA	3	-	NP	3	This study
EXNG525	77422	S	S	S	Not HLR	S	S	ND	NA	1596	WA-50	NA	325	-	NP	15	This study
EXNG526	63075	LS	S	S	Not HLR	S	S	ND	754	7363	WA-29	BPG-1	NA	NP	NP	-	This study
EXNG527	63076	LS	S	S	Not HLR	S	S	-	1245	7363	WA-29	BPG-1	NA	NP	NP	-	This study
EXNG530	93426	LS	S	S	Not HLR	S	S	-	NA	11428	WA-11	BPG-5	3	NP	NP	-	This study
EXNG532	63077	LS	S	S	Not HLR	S	S	-	1245	7363	WA-29	BPG-1	NA	NP	NP	-	This study
EXNG541	77423	PPNG	S	HLR	HLR	S	S	ND	NA	11965	WA-05	NA	NA	388	-	-	This study
EXNG545	77424	LS	S	S	Not HLR	S	S	-	NA	15331	WA-44	NA	NA	389	NP	82	This study
EXNG547	63078	LS	S	S	Not HLR	S	S	9920	754	7363	WA-29	BPG-1	380	NP	NP	-	This study
EXNG549	63079	LS	S	S	Not HLR	S	S	-	1245	7363	WA-29	BPG-1	NA	NP	NP	-	This study
EXNG552	77425	LS	S	HLR	Not HLR	S	S	2400	NA	7363	WA-31	NA	159	NP	NP	-	This study
EXNG553	63080	LS	S	S	Not HLR	S	S	18030	1245	7363	WA-29	BPG-1	NA	NP	NP	-	This study
EXNG554	77426	LS	S	S	Not HLR	S	S	5441, 14803	NA	8156	WA-36	NA	17	NP	NP	-	This study
EXNG555	63081	LS	S	S	Not HLR	S	S	8022	844	12046	WA-63	BPG-1	379	NP	NP	NP	This study

Isolate	PubMLST ID	PEN	CEF	CIP	TET	SPEC	AZI	NG-MAST	NG-STAR	MLST	WA type	BAP group	Ng_cgc_ 400	pST (conjugative)	pST (beta lactamase)	pST (cryptic)	Reference
EXNG556	63082	LS	S	S	HLR	S	S	9716	827	12042	WA-56	BPG-2	NA	-	NP	-	This study
EXNG563	77427	PPNG	S	LS	HLR	S	S	-	NA	9901	WA-26	NA	NA	-	-	-	This study
EXNG574	63083	LS	S	S	Not HLR	S	S	ND	NA	7363	WA-29	NA	380	NP	NP	-	This study
EXNG575	77428	LS	S	S	HLR	S	S	-	NA	8133	WA-53	NA	21	-	NP	32	This study
EXNG580	63084	LS	S	S	Not HLR	S	S	-	1245	7363	WA-29	BPG-1	NA	NP	NP	-	This study
EXNG583	63085	LS	S	S	Not HLR	S	S	-	754	12040	WA-59	BPG-1	380	NP	NP	-	This study
EXNG589	93425	S	S	S	Not HLR	S	S	ND	307	1596	WA-51	BPG-4	325	-	NP	15	This study
EXNG591	63086	LS	S	S	Not HLR	S	S	8022	844	12046	WA-63	BPG-1	379	NP	NP	NP	This study
EXNG592	63087	LS	S	S	Not HLR	S	S	-	1245	7363	WA-29	BPG-1	NA	NP	NP	33	This study
EXNG596	63088	LS	S	S	HLR	S	S	9716	827	12042	WA-56	BPG-2	381	-	NP	-	This study
EXNG601	93424	S	S	S	Not HLR	S	S	-	307	1596	WA-49	BPG-4	325	231	NP	-	This study
EXNG605	63089	LS	S	S	Not HLR	S	S	8022	844	12046	WA-63	BPG-1	379	NP	NP	NP	This study
EXNG606	63090	LS	S	S	Not HLR	S	S	-	754	7363	WA-29	BPG-1	380	NP	NP	-	This study
EXNG613	63091	LS	S	S	HLR	S	S	9716	827	12042	WA-56	BPG-2	381	-	NP	-	This study
EXNG615	94166	LS	S	S	Not HLR	S	R	5802	1034	9363	WA-13	BPG-5	3	NP	NP	-	This study
EXNG617	61271	PPNG	S	S	Not HLR	S	R	19509	270	1584	WA-38	BPG-3	33	10	-	8	This study
EXNG624	63092	LS	S	S	Not HLR	S	S	-	754	12040	WA-59	BPG-1	NA	NP	NP	-	This study
EXNG626	63093	LS	S	S	Not HLR	S	S	8022	844	12046	WA-63	BPG-1	379	NP	NP	NP	This study
EXNG635	77429	PPNG	S	HLR	HLR	S	S	ND	NA	10622	WA-04	NA	NA	390	-	-	This study
EXNG638	63094	LS	S	S	Not HLR	S	S	-	1245	7363	WA-29	BPG-1	NA	NP	NP	-	This study
EXNG640	63095	LS	S	S	Not HLR	S	S	-	1245	7363	WA-29	BPG-1	NA	NP	NP	-	This study
EXNG641	63096	LS	S	S	HLR	S	S	9716	827	12042	WA-56	BPG-2	NA	-	NP	-	This study
EXNG646	77430	PPNG	S	S	HLR	S	S	-	NA	10316	WA-57	NA	21	-	ND	-	This study
EXNG648	63097	LS	S	S	Not HLR	S	S	-	829	11648	WA-43	BPG-1	380	NP	NP	-	This study
EXNG650	63098	LS	S	S	Not HLR	S	S	4244	63	11428	WA-10	BPG-5	3	NP	NP	-	This study
EXNG658	93423	S	S	S	Not HLR	S	R	ND	1028	1596	WA-51	BPG-4	325	231	NP	-	This study
EXNG662	63099	CMRP	S	S	Not HLR	S	S	9716	827	12042	NA	NA	381	-	NP	33	This study
EXNG664	64011	LS	S	S	Not HLR	S	R	-	62	11428	WA-12	BPG-5	3	NP	NP	-	This study
EXNG675	63100	LS	S	S	Not HLR	S	S	8022	844	12046	WA-63	BPG-1	379	NP	NP	NP	This study
EXNG676	63101	LS	S	S	Not HLR	S	S	8022	844	12046	WA-63	BPG-1	379	NP	NP	NP	This study
EXNG685	61272	PPNG	S	S	Not HLR	S	R	19509	270	1584	WA-38	BPG-3	33	10	-	-	This study
EXNG687	63102	LS	S	S	Not HLR	S	S	-	1245	7363	WA-29	BPG-1	NA	NP	NP	-	This study
EXNG689	63103	LS	S	S	Not HLR	S	S	-	1245	7363	WA-29	BPG-1	380	NP	NP	-	This study
EXNG693	77431	PPNG	S	S	HLR	S	S	-	NA	8776	WA-07	NA	61	391	-	-	This study
EXNG697	63104	LS	S	S	Not HLR	S	S	-	1245	7363	WA-29	BPG-1	380	NP	NP	-	This study
EXNG702	93422	S	S	S	Not HLR	S	S	ND	307	1596	WA-51	BPG-4	325	-	NP	-	This study
EXNG711	63105	LS	S	S	Not HLR	S	S	18030	1245	7363	WA-29	BPG-1	NA	NP	NP	-	This study
EXNG716	61141	PPNG	S	S	Not HLR	S	R	19508	2168	1584	WA-38	BPG-3	33	10	5	8	This study
EXNG717	61142	PPNG	S	S	Not HLR	S	R	19508	2168	1584	WA-38	BPG-3	33	-	-	-	This study
EXNG718	63106	LS	S	S	Not HLR	S	S	8022	844	12046	WA-63	BPG-1	379	NP	NP	NP	This study
EXNG731	77432	PPNG	S	LS	Not HLR	S	S	-	NA	13266	WA-48	NA	25	295	-	-	This study
EXNG748	63107	LS	S	S	Not HLR	S	S	-	1245	7363	WA-29	BPG-1	NA	NP	NP	-	This study
EXNG750	63108	LS	S	S	Not HLR	S	S	-	1245	7363	WA-29	BPG-1	380	NP	NP	-	This study
EXNG752	63109	LS	S	S	Not HLR	S	S	-	1245	7363	WA-29	BPG-1	380	NP	NP	-	This study
EXNG755	63110	LS	S	S	Not HLR	S	S	-	1245	7363	WA-29	BPG-1	NA	NP	NP	-	This study
EXNG763	63111	LS	S	S	Not HLR	S	S	7343	1245	7363	WA-29	BPG-1	380	NP	NP	-	This study
EXNG765	63112	LS	S	S	Not HLR	S	S	-	NA	7363	WA-29	BPG-1	380	NP	NP	-	This study
EXNG769	63113	LS	S	S	Not HLR	S	S	-	1245	7363	WA-29	BPG-1	380	NP	NP	33	This study
EXNG771	63114	LS	S	S	HLR	S	S	9716	827	12042	WA-56	BPG-2	381	-	NP	-	This study
EXNG773	63115	LS	S	S	Not HLR	S	S	-	1245	7363	WA-29	BPG-1	380	NP	NP	-	This study
EXNG782	63116	LS	S	S	Not HLR	S	S	-	844	12045	WA-61	BPG-1	379	NP	NP	-	This study
EXNG793	63117	LS	S	S	Not HLR	S	S	8022	844	12046	WA-63	BPG-1	379	NP	NP	NP	This study
EXNG800	63119	LS	S	S	Not HLR	S	S	18030	1245	7363	WA-56	BPG-2	NA	NP	NP	-	This study
EXNG811	63118	LS	S	S	HLR	S	S	9716	827	12042	WA-29	BPG-2	381	-	NP	-	This study
EXNG821	77433	LS	S	S	Not HLR	S	S	14291	NA	11999	WA-08	NA	3	NP	NP	3	This study
EXNG827	61274	LS	S	S	Not HLR	S	R	7638	1029	1584	WA-38	BPG-3	33	348	NP	-	This study
EXNG839	94168	LS	S	HLR	Not HLR	S	R	15737	1487	9363	WA-15	BPG-5	3	NP	NP	3	This study
EXNG841	77434	PPNG	S	S	Not HLR	S	S	-	NA	11172	WA-37	NA	33	-	-	8	This study
EXNG844	63120	PPNG	S	HLR	HLR	S	S	ND	NA	1583	WA-34	NG	NA	361	-	-	This study
EXNG852	94169	LS	S	S	Not HLR	S	R	2992	NA	11864	WA-16	BPG-5	3	NP	NP	-	This study
EXNG855	63121	LS	S	S	Not HLR	S	S	8022	844	12046	NA	NA	379	NP	NP	NP	This study
EXNG856	63122	LS	S	S	HLR	S	S	9716	827	12042	WA-56	BPG-2	381	-	NP	-	This study
EXNG861	63123	LS	S	S	Not HLR	S	S	8022	844	12046	WA-63	BPG-1	379	NP	NP	NP	This study
EXNG863	63124	PPNG	S	HLR	HLR	S	S	ND	NA	7363	WA-29	NA	NA	NP	NP	-	This study
EXNG864	63125	LS	S	S	Not HLR	S	S	8022	844	12046	WA-63	BPG-1	379	NP	NP	NP	This study
EXNG873	77435	LS	S	S	R	S	S	ND	NA	1890	WA-68	NA	NA	NP	NP	-	This study
EXNG875	63126	LS	S	S	Not HLR	S	S	-	1245	7363	WA-29	BPG-1	380	NP	NP	-	This study
EXNG876	77436	PPNG	S	S	Not HLR	S	S	ND	NA	15608	WA-64	NA	NA	-	1	-	This study

Isolate	PubMLST ID	PEN	CEF	CIP	TET	SPEC	AZI	NG-MAST	NG-STAR	MLST	WA type	BAP group	Ng_cgc_400	pST (conjugative)	pST (beta lactamase)	pST (cryptic)	Reference
EXNG879	77437	CMRP	DS	HLR	Not HLR	S	S	14051	NA	7827	WA-42	NA	175	NP	NP	36	This study
EXNG897	77438	LS	S	HLR	Not HLR	S	S	-	NA	9362	WA-17	NA	3	NP	NP	3	This study
EXNG906	63127	LS	S	S	Not HLR	S	S	18030	1245	7363	WA-29	BPG-1	**NA**	NP	NP	-	This study
EXNG907	63128	PPNG	S	HLR	HLR	S	S	ND	NA	1583	WA-35	NG	NA	-	-	-	This study
EXNG912	63129	LS	S	S	Not HLR	S	S	-	754	7363	WA-29	BPG-1	380	NP	NP	-	This study
EXNG915	63130	LS	S	S	Not HLR	S	S	18030	1245	7363	WA-29	BPG-1	NA	NP	NP	27	This study
EXNG916	63131	LS	S	S	Not HLR	S	S	-	1245	7363	WA-29	BPG-1	380	NP	NP	-	This study
EXNG923	93421	LS	S	S	Not HLR	S	R	-	845	11428	WA-10	BPG-5	3	NP	NP	3	This study
EXNG924	77439	PPNG	S	HLR	HLR	S	S	ND	NA	8123	WA-28	NA	NA	-	-	-	This study
EXNG931	63132	LS	S	S	Not HLR	S	S	-	754	12040	WA-59	BPG-1	380	NP	NP	-	This study
EXNG932	63133	LS	S	S	HLR	S	S	9716	827	12042	WA-56	BPG-2	381	-	NP	-	This study
EXNG934	63134	LS	S	S	Not HLR	S	S	19520	844	12046	WA-63	BPG-1	379	NP	NP	-	This study
EXNG936	63135	LS	S	S	Not HLR	S	S	18030	1245	7363	WA-29	BPG-1	380	NP	NP	-	This study
EXNG939	77440	LS	S	R	Not HLR	S	S	-	NA	1583	WA-33	NA	NA	NP	NP	-	This study
EXNG941	63136	LS	S	S	Not HLR	S	S	8022	844	12046	WA-63	BPG-1	379	NP	NP	-	This study
EXNG942	77441	LS	S	HLR	HLR	S	S	-	NA	8775	WA-02	NA	NA	392	NP	-	This study
EXNG947	77442	LS	S	S	S	S	S	-	NA	1600	WA-78	NA	3	-	NP	-	This study
EXNG949	77443	LS	S	R	TRNG	S	S	-	NA	1583	WA-70	NA	29	393	-	-	This study
EXNG956	88783	LS	S	S	S	S	S	9897	301	11428	WA-10	BPG-5	3	NP	NP	3	This study
EXNG960	88784	LS	S	S	S	S	S	ND	754	1596	WA-75	BPG-1	380	NP	NP	-	This study
EXNG961	77444	PPNG	S	HLR	TRNG	S	S	11624	NA	7363	WA-67	NA	187	-	-	1	This study
EXNG962	77445	PPNG	S	HLR	TRNG	S	S	15199	NA	9903	WA-69	NA	111	394	-	8	This study
EXNG977	88785	LS	S	S	R	S	S	4244	63	11428	WA-10	BPG-5	3	NP	NP	-	This study
EXNG984	88786	LS	S	S	S	S	S	ND	754	1596	WA-75	BPG-1	380	NP	NP	-	This study
EXNG985	93420	LS	S	S	R	S	S	4244	63	11428	WA-10	BPG-5	3	NP	NP	-	This study
EXNG995	93419	LS	S	S	S	S	R	ND	845	11428	WA-10	BPG-5	3	NP	NP	-	This study
EXNG999	77446	LS	S	R	TRNG	S	S	16454	NA	6808	WA-74	NA	NA	-	NP	-	This study
EXNG1002	77447	LS	S	S	S	S	S	5441	NA	8156	WA-32	NA	17	NP	NP	8	This study
EXNG1005	77448	PPNG	S	R	TRNG	S	S	-	NA	7363	WA-30	NA	187	395	-	-	This study
EXNG1011	77449	LS	S	S	S	S	S	-	NA	13913	WA-73	NA	NA	NP	NP	9	This study
EXNG1012	77450	CMRP	DS	HLR	R	S	S	-	NA	1901	WA-21	NA	3	NP	NP	-	This study
EXNG1013	77451	LS	S	HLR	S	S	S	12140	NA	13480	WA-19	NA	25	-	NP	4	This study
EXNG1017	77452	PPNG	S	HLR	S	S	S	10354	NA	11191	WA-71	NA	106	-	5	17	This study
EXNG1026	77453	PPNG	S	R	TRNG	S	S	3379	NA	1587	WA-03	NA	187	14	-	9	This study
EXNG1034	88787	LS	S	S	S	S	S	4244	63	11428	WA-10	BPG-5	3	NP	NP	ND	This study
EXNG1036	77454	LS	S	S	S	S	S	-	NA	12045	WA-62	NA	379	NP	NP	ND	This study
EXNG1042	77455	LS	S	S	S	S	S	4186	NA	7359	WA-24	NA	177	NP	NP	-	This study
EXNG1052	88788	LS	S	S	S	S	S	18030	1245	7363	WA-29	BPG-1	380	NP	NP	-	This study
EXNG1053	77456	CMRP	DS	HLR	R	S	S	ND	NA	1600	WA-77	NA	402	NP	NP	4	This study
EXNG1058	77457	PPNG	S	HLR	TRNG	S	S	-	NA	11648	WA-72	NA	187	-	-	9	This study
EXNG1059	77458	CMRP	S	HLR	S	S	S	18031	NA	11981	WA-20	NA	3	NP	NP	3	This study
EXNG1063	77459	PPNG	S	HLR	TRNG	S	S	-	NA	8780	WA-76	NA	21	396	ND	32	This study
EXNG1066	77460	LS	S	S	S	S	S	13892	NA	1580	WA-66	NA	3	NP	NP	3	This study
M78932	61144	-	-	-	-	-	-	6747	1035	1584	NA	BPG-3	33	10	-	-	This study
M78933	61145	-	-	-	-	-	-	6747	270	1584	NA	BPG-3	33	-	-	-	This study
M78934	61146	-	-	-	-	-	-	6747	1036	1584	NA	BPG-3	33	-	-	8	This study
M78935	61147	-	-	-	-	-	-	6747	1036	1584	NA	BPG-3	33	10	-	-	This study

Supplementary Table 4. List of 970 loci used for the SNP-phylogenies in this study.

Locus	Locus	Locus	Locus	Locus	Locus	Locus	Locus	Locus	Locus	Locus	Locus	Locus	Locus	Locus	Locus	Locus	Locus	Locus	Locus	Locus	Locus	Locus	Locus
NEIS0009	NEIS0145 (rpsN)	NEIS0217	NEIS0340 (fetB2)	NEIS0470	NEIS0547	NEIS0630	NEIS0703	NEIS0782	NEIS0947	NEIS1106	NEIS1197	NEIS1284	NEIS1375	NEIS1457	NEIS1528	NEIS1645	NEIS1762	NEIS1846	NEIS1936	NEIS2046 (hemk)	NEIS2141	NEIS2633	NEIS2733
NEIS0014	NEIS0146 (rpsH)	NEIS0218	NEIS0344	NEIS0473	NEIS0549	NEIS0631	NEIS0704	NEIS0789	NEIS0948	NEIS1108	NEIS1198	NEIS1285	NEIS1376	NEIS1460	NEIS1530	NEIS1647	NEIS1763	NEIS1847 (rplU)	NEIS1937	NEIS2047	NEIS2143	NEIS2634	NEIS2734
NEIS0015 (glmU)	NEIS0147 (rplF)	NEIS0230	NEIS0346	NEIS0475	NEIS0550	NEIS0634	NEIS0705	NEIS0790	NEIS0958	NEIS1110	NEIS1200	NEIS1286	NEIS1378	NEIS1461	NEIS1535	NEIS1648	NEIS1765	NEIS1850 (rpmG)	NEIS1938	NEIS2049	NEIS2144	NEIS2635	NEIS2746
NEIS0018	NEIS0148 (rplR)	NEIS0237	NEIS0347	NEIS0476	NEIS0551	NEIS0635	NEIS0706	NEIS0791	NEIS0959	NEIS1111	NEIS1202	NEIS1287	NEIS1379	NEIS1466	NEIS1538	NEIS1649	NEIS1768	NEIS1851 (rpmB)	NEIS1949	NEIS2050	NEIS2146	NEIS2637	NEIS2748
NEIS0019	NEIS0149 (rpsE)	NEIS0238 (nuoB)	NEIS0348	NEIS0477	NEIS0552 (rpsO)	NEIS0636	NEIS0707	NEIS0793	NEIS0963	NEIS1112	NEIS1203	NEIS1288	NEIS1380	NEIS1471	NEIS1544	NEIS1650	NEIS1769 (ArsR)	NEIS1852	NEIS1952	NEIS2051	NEIS2147	NEIS2641	NEIS2749
NEIS0020 (pilB/msrAB)	NEIS0150 (rpmD)	NEIS0239	NEIS0349 (fHbp)	NEIS0480	NEIS0553	NEIS0637 (rnc)	NEIS0709	NEIS0800	NEIS0980	NEIS1114	NEIS1204	NEIS1290 (gatC)	NEIS1382	NEIS1473	NEIS1545	NEIS1651	NEIS1770	NEIS1853	NEIS1953	NEIS2052	NEIS2148 (pgk)	NEIS2642	NEIS2750
NEIS0040	NEIS0151 (rplO)	NEIS0240	NEIS0350 (fba)	NEIS0482	NEIS0554	NEIS0638	NEIS0711	NEIS0801	NEIS0981	NEIS1115	NEIS1205	NEIS1292	NEIS1383	NEIS1474	NEIS1546	NEIS1654	NEIS1772	NEIS1854	NEIS1954	NEIS2053	NEIS2149	NEIS2648	NEIS2751
NEIS0046 (rfbA)	NEIS0152	NEIS0241	NEIS0351	NEIS0484	NEIS0556	NEIS0639	NEIS0712	NEIS0812	NEIS0995	NEIS1116	NEIS1210	NEIS1293	NEIS1386	NEIS1475	NEIS1550	NEIS1655	NEIS1780	NEIS1855	NEIS1958	NEIS2055 (hldC)	NEIS2151	NEIS2650	NEIS2752
NEIS0047 (rfbB)	NEIS0153 (infA)	NEIS0242	NEIS0352	NEIS0498	NEIS0557	NEIS0640	NEIS0714	NEIS0814	NEIS1005	NEIS1125	NEIS1212	NEIS1294	NEIS1389	NEIS1477	NEIS1558	NEIS1674	NEIS1781	NEIS1857	NEIS1968	NEIS2057	NEIS2198 (opcA)	NEIS2652	NEIS2753
NEIS0048 (galE)	NEIS0155 (rpsM)	NEIS0245	NEIS0353	NEIS0500	NEIS0561	NEIS0641	NEIS0720	NEIS0818	NEIS1010	NEIS1128	NEIS1213	NEIS1295	NEIS1392	NEIS1480	NEIS1559	NEIS1675	NEIS1782	NEIS1867	NEIS1971	NEIS2058	NEIS2340	NEIS2658	NEIS2754
NEIS0059	NEIS0156 (rpsK)	NEIS0247	NEIS0354	NEIS0501	NEIS0563	NEIS0642	NEIS0721 (pilT2)	NEIS0819	NEIS1011	NEIS1129	NEIS1214	NEIS1296	NEIS1393	NEIS1481	NEIS1560	NEIS1676 (bioF)	NEIS1783	NEIS1870	NEIS1979	NEIS2059	NEIS2365	NEIS2659	NEIS2755
NEIS0071	NEIS0157 (rpsD)	NEIS0252	NEIS0355	NEIS0502	NEIS0566	NEIS0643	NEIS0722	NEIS0820	NEIS1013	NEIS1130	NEIS1216 (panD)	NEIS1298	NEIS1396 (fumC)	NEIS1483	NEIS1564	NEIS1677	NEIS1785	NEIS1871	NEIS1980	NEIS2060	NEIS2366	NEIS2663	NEIS2756
NEIS0072	NEIS0158	NEIS0253	NEIS0356	NEIS0503	NEIS0567	NEIS0644	NEIS0723	NEIS0823	NEIS1015 (abcZ)	NEIS1134	NEIS1218 (kdsA)	NEIS1299	NEIS1397	NEIS1485	NEIS1568	NEIS1680 (speB)	NEIS1786	NEIS1872	NEIS1981	NEIS2062	NEIS2367	NEIS2664	NEIS2757
NEIS0073	NEIS0159 (rplQ)	NEIS0254	NEIS0358	NEIS0504	NEIS0570	NEIS0645	NEIS0726	NEIS0839	NEIS1017	NEIS1135	NEIS1219	NEIS1300	NEIS1398	NEIS1486	NEIS1569	NEIS1682 (speA)	NEIS1787	NEIS1873	NEIS1982	NEIS2066 (cybB)	NEIS2375 (tatA/E)	NEIS2665	NEIS2758
NEIS0097	NEIS0160	NEIS0255	NEIS0361	NEIS0505	NEIS0571	NEIS0647	NEIS0730	NEIS0899 (lst)	NEIS1021	NEIS1136	NEIS1220 (eno)	NEIS1303	NEIS1400	NEIS1487	NEIS1570	NEIS1686	NEIS1788 (anmK)	NEIS1874	NEIS1984	NEIS2068	NEIS2381	NEIS2668	NEIS2760
NEIS0102	NEIS0162	NEIS0256 (xseB)	NEIS0374	NEIS0506	NEIS0572	NEIS0649	NEIS0731	NEIS0900	NEIS1022 (fbp)	NEIS1137	NEIS1221	NEIS1305	NEIS1407	NEIS1488	NEIS1571	NEIS1687	NEIS1807	NEIS1883	NEIS1998	NEIS2069	NEIS2399	NEIS2669	NEIS2761
NEIS0105	NEIS0166 (dadA)	NEIS0257	NEIS0375	NEIS0507	NEIS0574	NEIS0650	NEIS0732	NEIS0901	NEIS1026	NEIS1139	NEIS1222	NEIS1306	NEIS1410	NEIS1489	NEIS1578	NEIS1692	NEIS1808	NEIS1890	NEIS1999	NEIS2075	NEIS2405	NEIS2670	NEIS2762
NEIS0106	NEIS0167	NEIS0258	NEIS0379	NEIS0508	NEIS0575	NEIS0653	NEIS0735	NEIS0902	NEIS1027 (folB)	NEIS1140	NEIS1231	NEIS1307	NEIS1411	NEIS1490	NEIS1579	NEIS1699	NEIS1810 (aroE)	NEIS1891	NEIS2000	NEIS2076	NEIS2446	NEIS2673	NEIS2764
NEIS0107	NEIS0168 (lpxA)	NEIS0259	NEIS0385	NEIS0509	NEIS0576	NEIS0657 (rlpB)	NEIS0736	NEIS0905 (pip)	NEIS1029	NEIS1142	NEIS1232	NEIS1312	NEIS1412	NEIS1491	NEIS1581	NEIS1703	NEIS1811 (mtgA)	NEIS1896	NEIS2001	NEIS2077	NEIS2447	NEIS2674	NEIS2766
NEIS0109	NEIS0170	NEIS0261	NEIS0390	NEIS0512	NEIS0577	NEIS0659	NEIS0738	NEIS0906	NEIS1031	NEIS1143	NEIS1233	NEIS1314	NEIS1414	NEIS1492 (acnB)	NEIS1589	NEIS1704	NEIS1812 (yhbG)	NEIS1898	NEIS2002	NEIS2078	NEIS2448	NEIS2675	NEIS2767
NEIS0117 (secE)	NEIS0171 (lpxD)	NEIS0269	NEIS0392	NEIS0514	NEIS0578	NEIS0663	NEIS0739	NEIS0907	NEIS1034	NEIS1145	NEIS1237 (cmk)	NEIS1315	NEIS1415 (recX)	NEIS1494	NEIS1590	NEIS1720	NEIS1813 (ostA)	NEIS1899	NEIS2003	NEIS2079	NEIS2454	NEIS2676	NEIS2768
NEIS0118 (nusG)	NEIS0172	NEIS0270	NEIS0396 (pglD)	NEIS0515	NEIS0580 (argH)	NEIS0664	NEIS0741	NEIS0910	NEIS1035	NEIS1148	NEIS1239	NEIS1317	NEIS1419	NEIS1495	NEIS1591	NEIS1721	NEIS1814 (lptC)	NEIS1905	NEIS2004	NEIS2080 (rpsB)	NEIS2458	NEIS2677	NEIS2769
NEIS0119 (rplK)	NEIS0173	NEIS0271	NEIS0404	NEIS0516	NEIS0581 (galU)	NEIS0665	NEIS0743 (pgm)	NEIS0911	NEIS1036	NEIS1150	NEIS1240	NEIS1318	NEIS1423	NEIS1496	NEIS1592	NEIS1722	NEIS1815 (kdsC)	NEIS1906	NEIS2007	NEIS2081	NEIS2477 (drg)	NEIS2678	NEIS2772
NEIS0120 (rplA)	NEIS0174	NEIS0273	NEIS0405	NEIS0517	NEIS0583	NEIS0667	NEIS0744	NEIS0912	NEIS1037	NEIS1154	NEIS1242	NEIS1319 (hscB)	NEIS1425	NEIS1497	NEIS1593	NEIS1724	NEIS1816 (kpsF)	NEIS1907	NEIS2008	NEIS2108	NEIS2485	NEIS2680	NEIS2789
NEIS0121 (rplJ)	NEIS0175	NEIS0274	NEIS0406	NEIS0518	NEIS0585 (ntpA)	NEIS0669	NEIS0745	NEIS0913	NEIS1038	NEIS1155	NEIS1244 (ndk)	NEIS1320	NEIS1429	NEIS1498	NEIS1595	NEIS1725	NEIS1818 (talA)	NEIS1908	NEIS2010	NEIS2110	NEIS2488 (yfcA)	NEIS2683	NEIS2852
NEIS0122 (rplL)	NEIS0176	NEIS0288	NEIS0407	NEIS0522	NEIS0610 (apaH)	NEIS0672	NEIS0746	NEIS0915	NEIS1039	NEIS1157	NEIS1246	NEIS1326 (pgi1)	NEIS1431	NEIS1499	NEIS1599	NEIS1727 (ackA2)	NEIS1820	NEIS1909	NEIS2022	NEIS2113	NEIS2519	NEIS2684	NEIS2910
NEIS0123	NEIS0177	NEIS0289	NEIS0409 (pilP)	NEIS0523	NEIS0611	NEIS0674 (rpmI)	NEIS0747	NEIS0917	NEIS1040	NEIS1159	NEIS1248	NEIS1328	NEIS1432	NEIS1500	NEIS1600	NEIS1737	NEIS1822	NEIS1910	NEIS2023	NEIS2114	NEIS2522	NEIS2686	NEIS2936
NEIS0125 (rpsL)	NEIS0178	NEIS0304 (msbA)	NEIS0410 (pilO)	NEIS0528	NEIS0612	NEIS0676	NEIS0748	NEIS0919	NEIS1066	NEIS1164	NEIS1252	NEIS1329	NEIS1433	NEIS1501	NEIS1601	NEIS1739	NEIS1825	NEIS1911	NEIS2024	NEIS2116	NEIS2526	NEIS2690	
NEIS0126 (rpsG)	NEIS0182	NEIS0309	NEIS0414	NEIS0530	NEIS0613	NEIS0678 (vsr)	NEIS0751	NEIS0920	NEIS1067	NEIS1168	NEIS1256	NEIS1330	NEIS1438	NEIS1502	NEIS1603	NEIS1740	NEIS1826	NEIS1912	NEIS2025	NEIS2119	NEIS2530	NEIS2696	
NEIS0127	NEIS0183	NEIS0311	NEIS0418	NEIS0531 (rplS)	NEIS0614	NEIS0682	NEIS0753	NEIS0921	NEIS1071	NEIS1172	NEIS1257 (rplI)	NEIS1331 (gdh)	NEIS1439	NEIS1503	NEIS1604	NEIS1741	NEIS1827	NEIS1913	NEIS2026	NEIS2120	NEIS2552	NEIS2704	
NEIS0132 (rplC)	NEIS0185	NEIS0312 (rpmF)	NEIS0419 (gcp)	NEIS0532	NEIS0617	NEIS0683	NEIS0755	NEIS0922	NEIS1072	NEIS1173	NEIS1258 (rpsR)	NEIS1347	NEIS1440 (rpiA)	NEIS1504	NEIS1608	NEIS1742	NEIS1828	NEIS1915	NEIS2029	NEIS2122	NEIS2553	NEIS2706	
NEIS0133 (rplD)	NEIS0186	NEIS0313	NEIS0421 (lpxL2)	NEIS0533	NEIS0619	NEIS0684	NEIS0757	NEIS0923	NEIS1073	NEIS1175	NEIS1260 (rpsF)	NEIS1348	NEIS1442	NEIS1507	NEIS1609 (folP)	NEIS1743	NEIS1832	NEIS1916	NEIS2034	NEIS2123	NEIS2558	NEIS2707	
NEIS0134 (rplW)	NEIS0190	NEIS0315	NEIS0422	NEIS0534 (rpsP)	NEIS0620 (maeA)	NEIS0685	NEIS0760	NEIS0925 (sdhC)	NEIS1077	NEIS1176	NEIS1261	NEIS1351 (lpxL)	NEIS1443 (dnaQ)	NEIS1508	NEIS1610	NEIS1745	NEIS1833	NEIS1917	NEIS2035	NEIS2128	NEIS2580	NEIS2719	
NEIS0136 (rpsS)	NEIS0195	NEIS0318	NEIS0426	NEIS0536	NEIS0621 (lpxK)	NEIS0687	NEIS0761	NEIS0927 (sdhA)	NEIS1078	NEIS1178	NEIS1262	NEIS1353 (fis)	NEIS1444	NEIS1509	NEIS1613	NEIS1746	NEIS1834	NEIS1918	NEIS2037 (rpsI)	NEIS2129	NEIS2581	NEIS2721	
NEIS0137 (rplV)	NEIS0196	NEIS0319 (rpmH)	NEIS0430	NEIS0537	NEIS0622	NEIS0688	NEIS0762	NEIS0928 (sdhB)	NEIS1081	NEIS1179	NEIS1267	NEIS1355	NEIS1445	NEIS1511	NEIS1614	NEIS1747	NEIS1837 (pgi2)	NEIS1921 (rpsU)	NEIS2038 (rplM)	NEIS2130	NEIS2583	NEIS2722	
NEIS0139 (rplP)	NEIS0197 (fur)	NEIS0320	NEIS0431	NEIS0540	NEIS0623	NEIS0689	NEIS0766	NEIS0929	NEIS1082	NEIS1182	NEIS1270	NEIS1357	NEIS1446	NEIS1514	NEIS1619 (rfaK)	NEIS1748	NEIS1838 (pilG)	NEIS1922	NEIS2039	NEIS2132	NEIS2585	NEIS2724	
NEIS0140 (rpmC)	NEIS0198	NEIS0321 (dnaN)	NEIS0432	NEIS0541	NEIS0624 (kdsB)	NEIS0693	NEIS0767 (adk)	NEIS0930 (gltA)	NEIS1087	NEIS1183	NEIS1275	NEIS1363	NEIS1447	NEIS1515	NEIS1625	NEIS1753	NEIS1839 (pilD)	NEIS1924 (regG)	NEIS2040	NEIS2133 (etfB)	NEIS2588	NEIS2726	
NEIS0141 (rpsQ)	NEIS0200	NEIS0331	NEIS0434	NEIS0542	NEIS0625	NEIS0696	NEIS0768	NEIS0932 (sucB)	NEIS1088	NEIS1189	NEIS1276	NEIS1370	NEIS1449	NEIS1516	NEIS1629	NEIS1755	NEIS1841	NEIS1931	NEIS2041	NEIS2134 (rfaC)	NEIS2592	NEIS2727	
NEIS0142 (rplN)	NEIS0204	NEIS0334	NEIS0435	NEIS0543	NEIS0626	NEIS0697	NEIS0775 (clpS)	NEIS0935 (sucC)	NEIS1090	NEIS1190	NEIS1279 (aceF)	NEIS1371	NEIS1453	NEIS1524 (gpm)	NEIS1639	NEIS1756	NEIS1842	NEIS1932	NEIS2043	NEIS2137 (gapA2)	NEIS2601	NEIS2729	
NEIS0143 (rplX)	NEIS0207	NEIS0337	NEIS0464	NEIS0545	NEIS0628	NEIS0699	NEIS0776	NEIS0936 (sucD)	NEIS1103	NEIS1191	NEIS1280 (lpd)	NEIS1373	NEIS1455	NEIS1525	NEIS1640	NEIS1759	NEIS1844 (pilF)	NEIS1933	NEIS2044	NEIS2139	NEIS2602	NEIS2730	
NEIS0144 (rplE)	NEIS0216	NEIS0339	NEIS0468	NEIS0546 (hisE)	NEIS0629	NEIS0702 (hfq)	NEIS0781	NEIS0942	NEIS1104	NEIS1196	NEIS1281	NEIS1374	NEIS1456 (rfaF)	NEIS1526	NEIS1643 (CcoO)	NEIS1760	NEIS1845	NEIS1934	NEIS2045	NEIS2140	NEIS2604	NEIS2731	

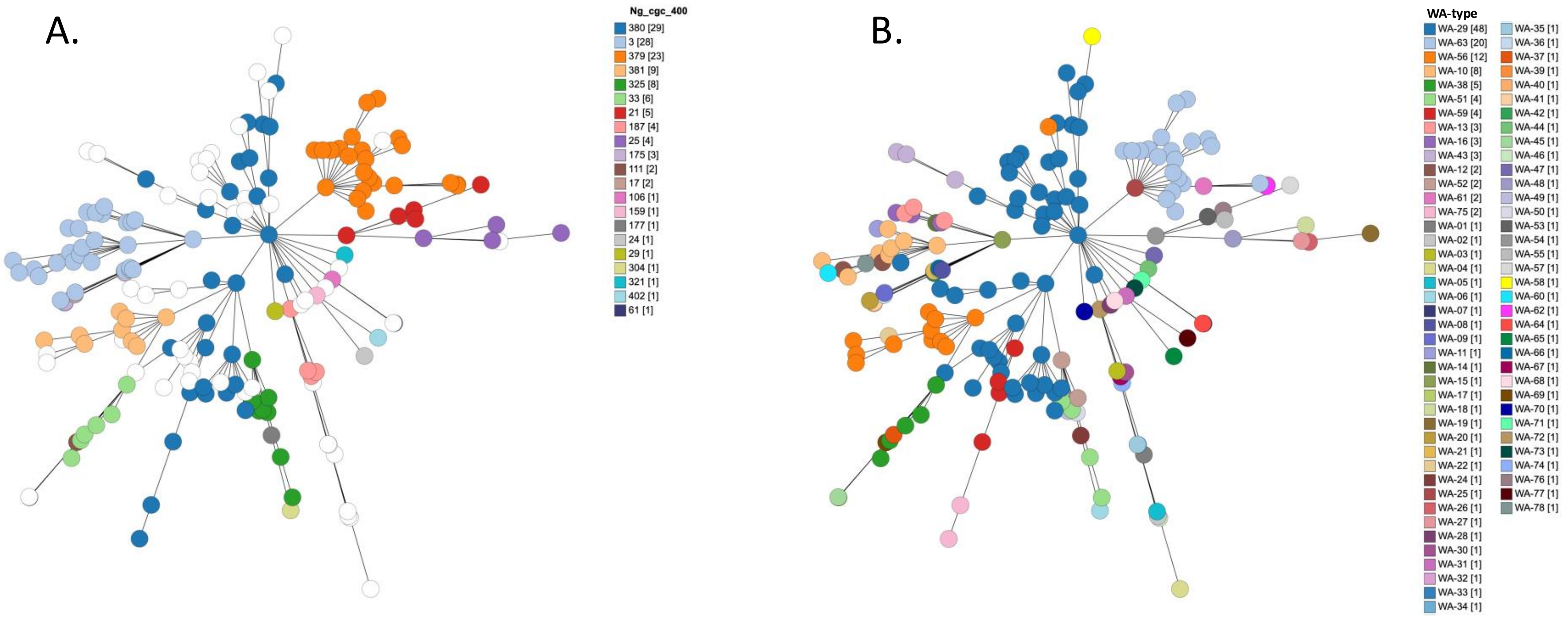

Supplementary Figure 1: Minimum spanning tree based on cgMLST labelled by Ng_cgc_400 groups (Panel A) and iPLEX® genotypes (Panel B).

Minimum spanning tree based on core genome allelic profiles of 181 *N. gonorrhoeae* isolates collected in Western Australia in 2017. Whole genome sequence data were compared using GrapeTree resulting in isolates with similar cgMLST allelic profiles forming clusters.

Panel A. The minimum spanning tree was generated using the cgMLST scheme (N. gonorrhoeae cgMLST v1.0) set at 400 or fewer locus threshold (25 Novemeber 2022). Core genome groups were coloured and named x [number of isolates]. Core genome groups with less than 10 isolates in PubMLST database are represented with white circles.

Panel B. The tree in Panel A now overlayed with iPLEX® genotypes. WA-types were coloured and named x[number of isolates].

Panel A: Recent recombinations

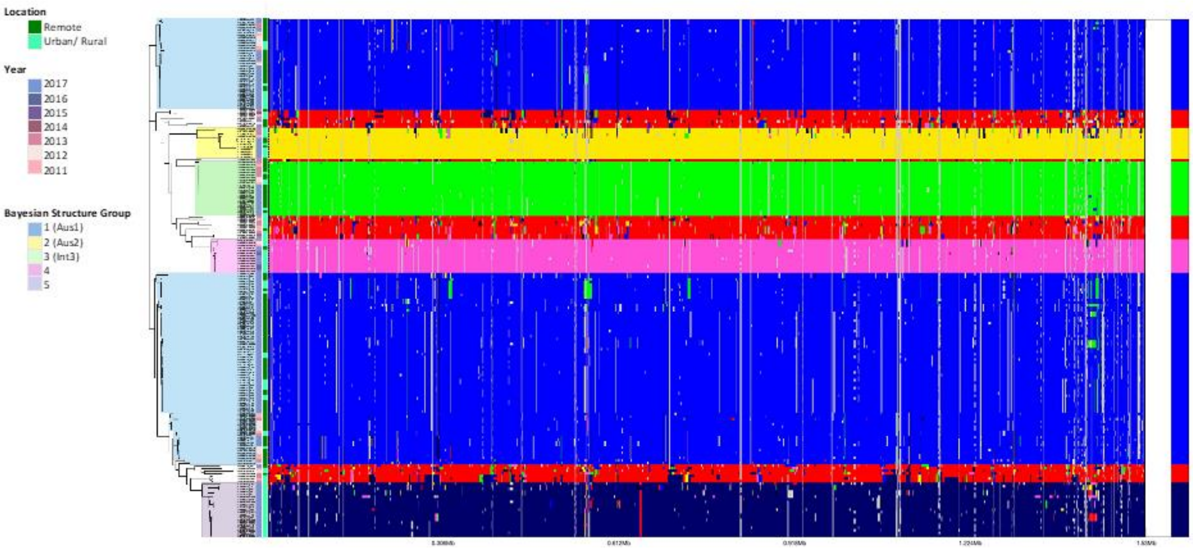

Panel B: Ancestral recombinations

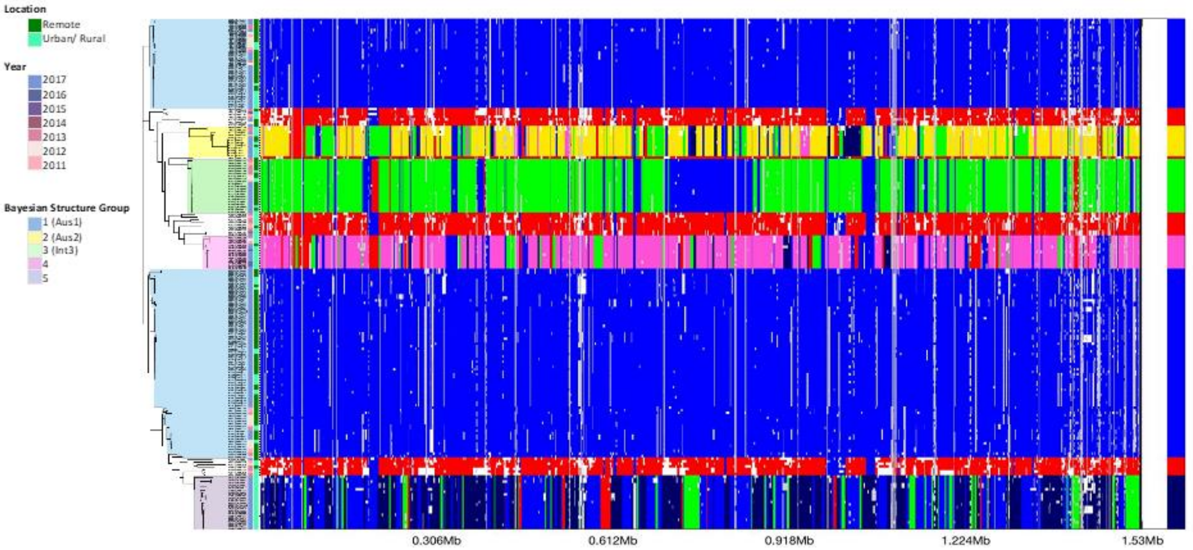

Supplementary Figure 2: fastGEAR results based on the core genome of 196 *N. gonorrhoeae* strains. The maximum likelihood phylogeny is shown in the left and coloured based on the identified BAPS structure groups and annotated with location and year of isolation

Panel A shows recent recombinations for the dataset. On the y-axis are the 196 sequences present in the alignment and on the x-axis the sequence positions. The annotation on the right side of the panel shows division of the strains into six structure groups (blue, yellow, green, red, pink and indigo). The sequences are coloured based on the ancestry of the sequences (black colour denotes recombination estimated to come from outside of any lineage in the data set). The red group is a collection of the most divergent isolates which is not biologically meaningful.

Panel B shows ancestral recombinations in the dataset. The recent recombinations are removed before the analysis of ancestral recombinations, and therefore the recent recombinations are shown as white gaps. the direction of ancestral recombinations is not formally identifiable using fastGEAR.
